# Effects of Different Paired-Set Configurations and Rest Intervals on Acute Volume Load, Repetitions, and Training Efficiency: A Systematic Review

**DOI:** 10.3390/jfmk11030370

**Published:** 2026-09-11

**Authors:** Kuei-Yu Chien, Tzu-Han Chan, Ting-Ru Lin, Tzu-Chen Lin

**Affiliations:** 1 Graduate Institute of Sports Science, National Taiwan Sport University, No. 250, Wenhua 1st Rd., Guishan Dist., Taoyuan City 33325, Taiwan; 1120201@ntsu.edu.tw; 2 Department of Athletic Training and Health, National Taiwan Sport University, Taoyuan City 33325, Taiwan; 1140601@ntsu.edu.tw; 3 College of Exercise and Health Sciences, National Taiwan Sport University, Taoyuan City 33325, Taiwan; mila19860302@gmail.com

**Keywords:** paired sets, supersets, resistance exercise, volume load, completed repetitions, rest interval, training efficiency

## Abstract

**Background:** Paired-set (PS) resistance exercise is widely used to increase training density; however, evidence-based guidance remains limited due to heterogeneous exercise sequences and rest configurations. This systematic review aimed to synthesize acute mechanical, training-efficiency, and perceptual responses across distinct PS configurations compared with traditional sets (TSs) in young resistance-trained adults. **Methods:** Eight databases were searched through 5 August 2026, for acute randomized crossover/parallel trials. PS protocols were categorized into PS-A (alternating exercises with preserved TS rest after each exercise), PS-B (interleaved exercises during recovery), and PS-C (modified post-pair rest). Study-level means and unstandardized percentage differences for volume load (VL), completed repetitions, training efficiency (VL accumulated per unit of time), and rating of perceived exertion (RPE) were synthesized descriptively. **Results:** Fifteen reports representing 14 studies (223 participants) were included, and all exhibited “some concerns” according to RoB 2. PS-A consistently elicited higher work-output point estimates (VL: 7.9–27.2%; repetitions: 6.3–26.7% vs. TS) with negligible changes in session duration. PS-B consistently reduced session duration by 44.8–50.0%, though repetitions and VL were often compromised depending on the exercise. PS-C responses were configuration-dependent; matched post-pair rest (PS-C_MPR_) decreased duration (39.7–46.9%) with variable VL (−8.2% to +30.1%) and higher RPE, whereas extended rest (PS-C_EPR_) showed distinct protocol-specific trade-offs between volume retention and training efficiency. **Conclusions:** Based on acute trials in young trained populations, PS configurations should be selected by session priority (PS-A for work capacity, PS-B for time constraints, PS-C for versatility). Given the consistent “some concerns” regarding risk of bias, these practical recommendations warrant caution.

## 1. Introduction

Resistance training is effective for increasing strength and power in general and athletic populations [1,2,3,4,5]. Variables such as training volume, rest interval between sets, loading, and exercise selection are closely associated with training outcomes [5,6]. Volume load (VL) is calculated as the mathematical product of sets, repetitions, and external load [1]. At matched relative load, the accumulation of volume load within a single session serves as a primary mechanical driver of resistance training adaptations [7,8,9]. Because volume load is a key determinant of training outcomes, and repetition capacity directly determines the volume load accumulated within a session, maintaining repetition performance across consecutive sets is essential. While longer inter-set rest intervals facilitate intra-session neuromuscular recovery and preserve volume load, they substantially prolong total session duration [6]. Perceived lack of time remains one of the most frequently reported barriers to regular resistance training [10,11], reflecting broader occupational and scheduling demands. Therefore, the development of efficient, time-saving training methods is warranted.

In resistance training, a paired set refers to a set-configuration strategy in which two distinct exercises are paired and executed alternately or consecutively, instead of completing all sets of one exercise before performing the next; these pairings can involve agonist–antagonist, upper–lower-body, or biomechanically similar exercise combinations [12,13]. PSs can be further categorized based on exercise selection, such as agonist–antagonist, upper–lower-body, unrelated muscle group, and same muscle group [14,15,16,17]. In addition to exercise selection, variables such as training load, repetitions, and inter-set rest intervals have inconsistent effects on volume load, completed repetitions, and training efficiency across PS protocols [14,18,19,20]. In the present review, consistent with terminology used in the prior literature [19], training efficiency is operationally defined as volume-load efficiency, calculated as volume load completed per unit of time, which increases with greater volume load, shortened session duration, or both. For consistency, the term training efficiency is used throughout this review to refer to this construct. Studies comparing PS with traditional sets (TSs) have reported inconsistent findings for volume load, repetition performance, and training efficiency, probably stemming from variations in protocol characteristics across studies [2,11]. First, exercise pairing may contribute to different acute responses [12,17]. Agonist–antagonist studies have reported favorable volume-load responses under some paired-set conditions [12,18,19]. Robbins et al. [19] investigated three sets of four-repetition maximum (RM) barbell bench presses and bent-over rows. In the PS group, a 2 min nominal rest interval was allocated between exercises, effectively accumulating to a 4 min recovery window before the same exercise was repeated, while a 2 min rest interval was implemented in the TS group. Total volume load, along with the volume load of the second and third sets, was higher in the PS condition, while session duration was the same (PS: 10 min vs. TS: 10 min) [19]. Results from upper–lower-body protocols have been less consistent: when upper–lower-body pairings or whole-body alternating sequences are implemented; acute work capacity can be impaired. For instance, Peña García-Orea et al. [13] reported fewer total squat repetitions under the paired condition, whereas bench press repetitions did not differ from TS, suggesting that performance decrements may be exercise-dependent when additional multi-joint work is performed during the recovery period. Second, the effective recovery duration before the same exercise is repeated may also be important [10]. Using bench press and bench pull at the same 4RM load, Robbins et al. [20] compared acute PS and TS protocols. When both protocols provided an identical 4 min recovery window before the same exercise was repeated, no significant difference in training volume was found between methods; however, the PS configuration reduced total training duration relative to TS (PS: 10 min vs. TS: 20 min) [20]. Third, other protocol characteristics, including the number of exercise pairs or total exercises [21], training load (e.g., heavy 4RM vs. moderate 10RM) [2], and set-termination criteria or proximity to failure (e.g., velocity-loss termination vs. momentary muscular failure) [13], may also contribute to the variability in acute outcomes. Consequently, comparing PS and TS without controlling for these critical modifiers can generate conflicting conclusions. Therefore, systematically examining these primary sources of heterogeneity across different PS designs is necessary.

Previous reviews have suggested that superset- or paired-set-based training can reduce session duration and improve training efficiency while generally maintaining volume load and repetition performance [11,22]. Supersets (or compound sets) typically denote the consecutive execution of two exercises targeting the same muscle group with minimal intervening rest, and Mang et al. [23] further highlighted that acute responses vary across superset configurations. However, these syntheses primarily categorized protocols by anatomical pairing (e.g., agonist–antagonist vs. alternate peripheral exercises) rather than categorizing protocols according to recovery distribution relative to the traditional-set comparator. Consequently, how different PS configurations influence acute training volume, repetition capacity, and training efficiency could not be adequately determined. Furthermore, there remains a lack of systematic research synthesizing how rest intervals and exercise combinations interact to shape acute volume load, repetitions, and session duration. The present review therefore aimed to systematically evaluate the effects of different paired-set configurations, categorized according to recovery distribution, on acute volume load, repetition output, and training efficiency during single-bout resistance training, offering practical prescription insights for resistance-trained adults seeking to optimize training under time-constrained conditions.

## 2. Materials and Methods

The present systematic review is reported in accordance with the PRISMA 2020 statement [24], with reporting additionally informed by the PERSiST guidance for sport and exercise science [25] and, for synthesis without meta-analysis, the SWiM reporting guidelines [26]. The review question, eligibility criteria, and primary outcomes were structured using the Population, Intervention, Comparator, Outcomes, and Study Design (PICOS) framework. This review was not prospectively registered, and no formal review protocol was prepared. Core eligibility criteria and primary outcomes were established a priori using the PICOS framework; the paired-set categories used for synthesis were developed after data extraction to organize the included protocols.

### 2.1. Search Strategy

A systematic literature search was conducted across eight electronic databases: PubMed/MEDLINE, SPORTDiscus (EBSCOhost), Scopus, Web of Science, CINAHL (EBSCOhost), Rehabilitation & Sports Medicine Source (EBSCOhost), Embase, and the Cochrane Central Register of Controlled Trials (CENTRAL). The final database search was completed on 5 August 2026, with no publication-date restriction. Database-specific controlled vocabulary and free-text terms were combined to capture resistance training and paired-set, superset, alternating-set, and related exercise configurations. English-language restrictions were applied where available; CENTRAL was searched without a language limiter. The complete database-specific search strategies, fields, filters, search dates, and numbers of retrieved records are provided in Supplementary Table S1.

Following database screening, the 12 included reports identified through electronic database searching served as seed references for supplementary citation searching. Two relevant reviews [22,23] were also used as seed references to broaden the identification of potentially eligible studies. Backward and forward citation searching was conducted using Citationchaser [27]. Records identified through citation searching were screened using the same eligibility criteria as database records. The search strategy and database-specific syntax were developed and checked by the review team, and all electronic database searches were executed by T.H.C.

### 2.2. Eligibility Criteria

Eligibility criteria were defined using the PICOS framework (Table 1). Studies were eligible if they included healthy, resistance-trained adults aged ≥18 years with at least 6 months of continuous resistance-training experience and a training frequency of ≥1 session per week. The 6-month threshold is consistent with the American College of Sports Medicine description of intermediate trainees as individuals with approximately 6 months of consistent resistance-training experience [7]. The ≥1-session-per-week criterion was used as a minimum exposure threshold rather than as a definition of training level; once-weekly minimal-dose resistance training has been shown to produce sustained strength adaptation over long-term follow-up [28]. Training status was additionally interpreted with reference to the participant classification framework proposed by McKay et al. [29], with Tier 2 (trained/developmental) representing the minimum trained category. Eligible interventions involved dynamic resistance exercise in which two exercises targeting different primary muscle groups were paired and performed alternately or consecutively. Each exercise was required to be performed for at least three sets to enable examination of acute fatigue accumulation and changes in volume load and completed repetitions across sequential sets within a session. Intervention pairing exercises targeting the same primary muscle group (e.g., pre-exhaustion or compound sets) were excluded because they did not match the paired-set structure examined in this review. Interventions involving tri-sets, circuits, or other configurations comprising three or more exercises performed in sequence were excluded because they did not meet the review definition of a paired-set condition involving two exercises. Traditional-set comparators used the same exercises, number of sets, and prescribed loads or relative intensities as the paired-set condition, allowing exercise sequence and rest structure to be compared.

The primary outcomes comprised acute volume load, completed repetitions, and training efficiency. Studies utilizing either repetition-maximum protocols or velocity-loss criteria to terminate sets were eligible, with these methods recorded as protocol characteristics reflecting different proximities to muscular failure. Eligible study designs comprised randomized crossover and randomized parallel-group trials examining acute resistance-exercise responses, whereas longitudinal interventions and non-randomized studies were excluded. Finally, eligibility was restricted to peer-reviewed English-language full-text reports. Conference abstracts were excluded because they did not provide sufficient protocol and outcome information for extraction.

### 2.3. Study Selection Process

Database records were exported to Zotero for duplicate removal and subsequently imported into Rayyan for screening. Titles and abstracts were independently screened by two reviewers (T.H.C. and T.R.L.), followed by independent full-text assessment of potentially eligible reports. Disagreements between the two reviewers were resolved through discussion; if consensus could not be reached, a third reviewer (K.Y.C.) was consulted to arbitrate and establish a final decision. Reasons for exclusion at the full-text stage were recorded, and the study-selection process is presented in the PRISMA flow diagram. Potentially overlapping reports were assessed by comparing the reported author group, ethics-approval information, participant characteristics, and intervention, comparator, and testing procedures. Reports were classified as companion reports when these details indicated that they arose from the same underlying study. Companion reports were retained when they contributed distinct outcome information but were linked as one underlying study and were not treated as independent replications. Outcome data were extracted from the report in which each outcome was presented; when companion reports reported different sample sizes and the exact degree of participant overlap could not be determined, participant-level summaries used the sample from the report contributing the outcome most directly relevant to the prespecified primary outcomes.

### 2.4. Data Extraction and Synthesis

Data were extracted independently by T.H.C. and T.R.L. and cross-checked; disagreements were resolved through discussion, with K.Y.C. consulted when consensus could not be reached. Extracted information included study design, participant characteristics, loading, exercises, pairing and order, set structure, within-pair and post-pair rest, recovery before repeating the same exercise, and the outcomes. Numerical data were taken directly from article text and tables; values were not digitized from figures or imputed, and study authors were not contacted for missing data. Outcomes were retained at the exercise, set, or session level reported for each paired-set condition and TS comparator. Session durations reported directly by the original studies were distinguished from durations calculated from protocol information or reported only approximately. Recovery before repeating the same exercise was recorded directly when available; otherwise, the minimum protocol-specified interval was calculated from within-pair and post-pair rest. Because intervening-exercise and transition times were usually unavailable, these intervals were treated as minimum or nominal rather than exact end-to-start recovery. Volume load was defined as sets × repetitions × external load. Completed repetitions and training efficiency were retained as reported, whereas session duration, an intervention-level parameter, was retained as reported to contextualize training efficiency. Graphical or narrative efficiency findings are described without reconstructing exact values. Secondary outcomes are summarized as reported by the original studies. Numerical contrasts were calculated only when the required scalar values were available; other unreported quantities were not reconstructed, and secondary outcomes were not used to determine the primary synthesis pattern.

Findings were synthesized descriptively around volume load, completed repetitions, and training efficiency. Meta-analysis was not undertaken because the materially different paired-set configurations and comparator rest structures did not support an interpretable common effect, multiple exercise-, condition-, and set-level results within the same underlying studies were dependent, and the paired variance required to estimate the precision of most crossover comparisons was unavailable [30,31]. Multiple results from a single underlying study are therefore presented separately but are not treated as independent studies.

Interpretation emphasized reported condition means and unstandardized mean differences at the exercise, set, or session level rather than counts of statistically significant results. Because PS and TS outcomes within each comparison were expressed on the same measurement scale, differences were retained in their original units to preserve direct interpretation [32]. When scalar means were available, the unstandardized mean difference was calculated as PS minus TS. For crossover trials, the difference between the two condition means provides the same mean-difference point estimate as the mean of the participant-level paired differences, although paired variability is required in order to estimate its standard error and confidence interval [31]. The relative difference was calculated as (PS − TS)/TS × 100 and used to describe the magnitude of each difference relative to its corresponding TS value. Relative differences were treated as descriptive presentation metrics rather than standardized effect sizes or pooled estimates. For crossover comparisons, 95% confidence intervals were not calculated when the standard deviation of the paired differences or the within-participant correlation was unavailable, and no correlation was assumed. For the parallel-group study by Peña García-Orea et al. [13], unstandardized mean differences and 95% confidence intervals were calculated from the reported group sample sizes, means, and standard deviations using Welch’s unequal-variance method [33].

For the configuration-level descriptive summary, one representative relative difference was assigned to each underlying study for each configuration–outcome combination. When a study contributed a single eligible scalar estimate, that estimate was used directly; when multiple eligible scalar estimates were available within the same study for the same review-defined configuration and outcome, their unweighted median was used so that each underlying study contributed once. For presentation in the configuration-level summary, an unweighted median of these study representatives was reported when at least three underlying studies contributed eligible scalar estimates; with fewer than three studies, the available estimates were presented without a central summary. The full available-estimate range retained the lowest and highest scalar estimates at their reported analysis levels to show the complete observed span. These summaries were descriptive, were not weighted by study size or precision, and were not interpreted as pooled effects.

Paired-set conditions were categorized after data extraction according to exercise sequence and rest structure to distinguish how each configuration redistributed exercise and recovery relative to its TS comparator. PS-A alternated two exercises while preserving the rest used after each exercise in TS. PS-B placed the alternate exercise within the recovery period before the same exercise was repeated. PS-C performed the exercises consecutively with no prescribed inter-exercise rest, or only transition time, followed by post-pair rest. PS-C was further classified as matched post-pair rest (PS-C_MPR_), when post-pair rest matched TS, or extended post-pair rest (PS-C_EPR_), when it exceeded TS. Comparisons involving different TS rest structures were retained separately because the interpretation of a paired-set contrast depends on the recovery provided by its comparator. Multiple paired-set conditions or TS comparators within a study are therefore described separately.

**Figure 1:**
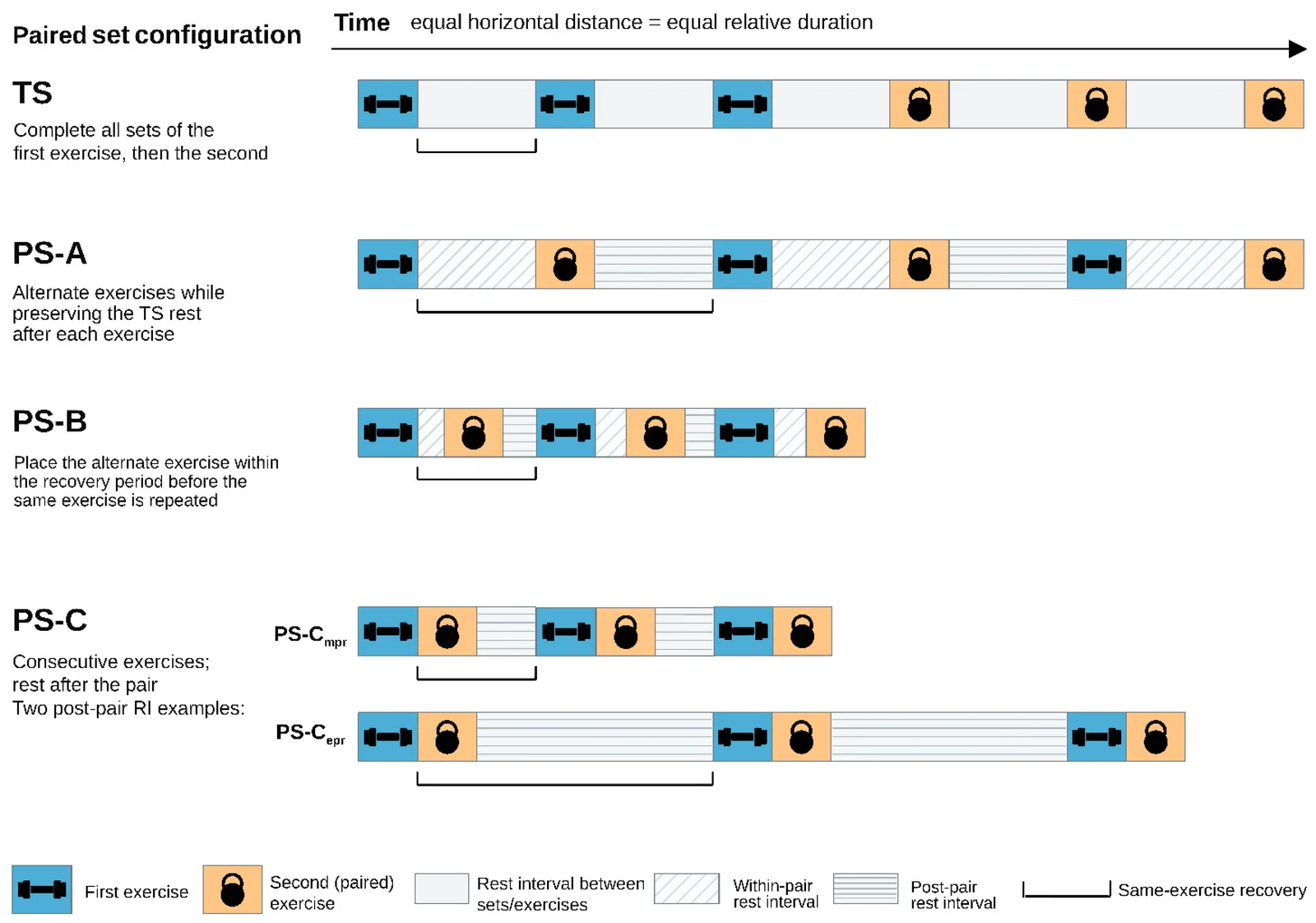
Schematic representation of paired-set configurations and rest-interval structures. The schematic illustrates how exercise order and recovery are distributed in traditional sets (TSs) and the three review-defined paired-set (PS) configurations. PS-A alternates two exercises while preserving the rest used after each exercise in the TS, thereby lengthening recovery before the same exercise is repeated. PS-B places the alternate exercise within the recovery period before the same exercise is repeated. PS-C performs the two exercises consecutively with no prescribed inter-exercise rest, or only transition time, followed by post-pair rest. PS-C is further classified as matched post-pair rest (PS-C_MPR_), in which post-pair rest matches TS, and extended post-pair rest (PS-C_EPR_), in which post-pair rest exceeds TS. Horizontal distances are schematic and do not represent absolute durations; actual rest intervals varied across studies. Brackets indicate recovery before the same exercise is repeated. Abbreviations: RI, rest interval; TS, traditional set; PS, paired set; MPR, matched post-pair rest; EPR, extended post-pair rest.

Protocols were interpreted according to exercise pairing, session scale, recovery before repeating the same exercise, and result-level RoB 2. Statistical significance is reported when relevant but was not used to infer equivalence, a favorable trend, or practical superiority without an estimate and its available uncertainty. The paired-set configurations and rest-interval structures are illustrated in Figure 1.

### 2.5. Risk of Bias and Methodological Quality Assessment

Two complementary approaches were used to evaluate the included evidence. The Cochrane Risk of Bias 2 (RoB 2) tool was the primary appraisal of bias in the specific results contributing to the synthesis, whereas a modified Physiotherapy Evidence Database (PEDro) scale provided complementary report-level methodological quality information [34,35,36,37]. The standard RoB 2 tool was used for the randomized parallel-group trial, and the crossover variant was used for randomized crossover trials, including the additional domain addressing period and carryover effects. The effect of interest was the effect of assignment to intervention. Outcome-specific assessments were completed for volume load, completed repetitions, and training efficiency according to the RoB 2 algorithms. For the modified PEDro assessment, item 1 (eligibility criteria) was recorded descriptively but excluded from the total score, and items 5–7 were omitted because participant, therapist, and assessor blinding were not feasible in these supervised resistance-training protocols. The modified score therefore comprised items 2–4 and 8–11, with a maximum score of 7. Modified PEDro total scores were reported descriptively; no categorical quality labels or cutoff thresholds were applied.

T.H.C. and T.R.L. independently completed the RoB 2 and modified PEDro assessments, with disagreements resolved by consensus or consultation with K.Y.C. Pre-consensus inter-rater agreement was calculated separately for each appraisal using exact percentage agreement and unweighted Cohen’s κ. RoB 2 and modified PEDro were interpreted as complementary assessments and were not combined into a single score or judgment.

## 3. Results

### 3.1. Study Selection

**Figure 2:**
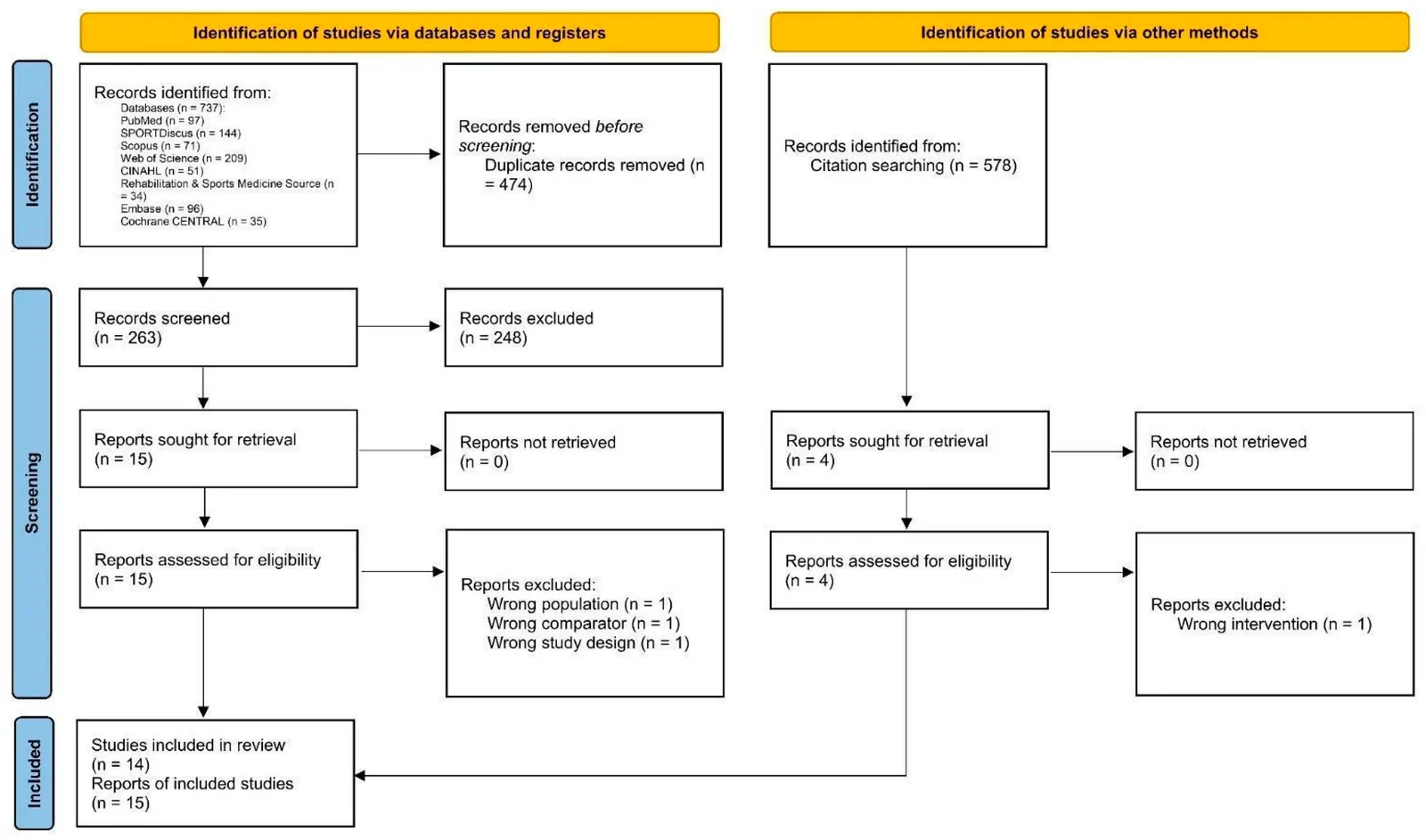
Preferred Reporting Items for Systematic Reviews and Meta-analyses (PRISMA) flow diagram.

The search strategy yielded 737 records. After the removal of 474 duplicate records, the titles and abstracts of 263 records were screened (Figure 2). From these, 248 records were excluded because they were not relevant to paired-set and traditional-set resistance training, resulting in 15 reports for full-text assessment. Three reports were subsequently excluded: one because of an ineligible population [38], one because of an ineligible comparator [39], and one because of an ineligible study design (a book review rather than a primary research report), leaving 12 reports included through database searching. Citation searching identified 578 additional records, from which four reports underwent full-text assessment; one was excluded because of an ineligible intervention [40], leaving three reports included through citation searching. Reasons for full-text exclusion are provided in Supplementary Table S2 [38,39,40,41]. Two reports were considered likely to be companion reports from the same underlying study: Paz et al. [42] and Paz et al. [17]. Because both reports described the same ethics approval, shared overlapping authorship, and implemented substantially similar exercise protocols, they were classified as companion reports from a single underlying trial, even though differing sample sizes (13 vs. 22 participants) precluded determining the exact extent of participant overlap. The reports were retained separately because they provided different outcome data, but they were counted as one underlying study. Therefore, 15 reports representing 14 underlying studies (12 from database searching and 3 from citation searching) were included in the systematic review. The study-selection process is shown in the PRISMA flow diagram (Figure 2).

### 3.2. Study Characteristics

The 14 underlying studies included 223 participants, comprising 208 men and 15 women. This total accounted for the companion reports by Paz et al. [17] and Paz et al. [42]. Because both reports described the same underlying trial, only the sample of 22 participants from Paz et al. [17] (the report contributing the training-performance outcomes most directly aligned with the review’s primary outcomes) was used when calculating the total participant count. To avoid double-counting, the 13 participants from the companion report were not added separately. Sample sizes across the 14 underlying studies ranged from 10 to 29 participants, and reported mean ages ranged from 20.0 to 28.1 years. Thirteen studies used randomized crossover designs, and one used a randomized parallel-group design; in the latter, reported by Peña García-Orea et al. [13], the four testing sessions represented repeated acute assessments at different relative loads and were treated as repeated observations within a single underlying study rather than as independent studies or a longitudinal intervention. The evidence was derived predominantly from young, resistance-trained men; Andersen et al. [14] was the only report that included women.

All studies evaluated acute resistance-exercise responses rather than longitudinal training adaptations. Most protocols used three sets per exercise, with loading prescribed using repetition-maximum, percentage-based, or velocity-loss approaches. Agonist–antagonist pairings predominated, although upper–lower-body and mixed whole-body pairings were also represented. Detailed participant, protocol, rest-interval, and outcome characteristics are presented in Table 2, and the three paired-set configurations are illustrated in Figure 1.

### 3.3. Risk of Bias and Methodological Quality

#### 3.3.1. Risk of Bias via RoB 2

**Figure 3:**
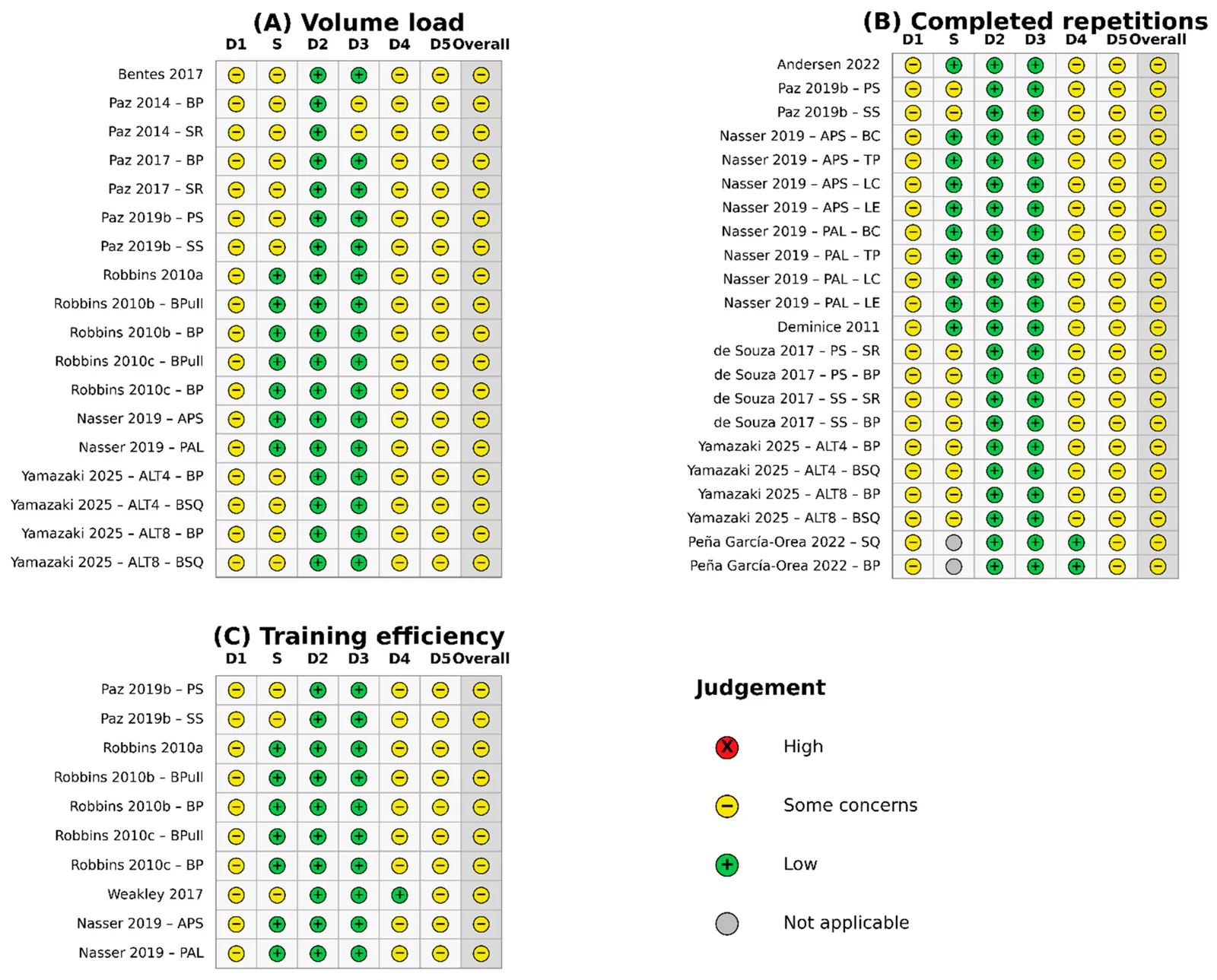
Risk of Bias 2 Assessments by Primary Outcome Category. Note. Panels present RoB 2 result-level judgments for (**A**) volume load, (**B**) completed repetitions, and (**C**) training efficiency. D1 = bias arising from the randomization process; S = bias arising from period and carryover effects; D2 = bias due to deviations from intended interventions; D3 = bias due to missing outcome data; D4 = bias in measurement of the outcome; D5 = bias in selection of the reported result. Red X = high risk; yellow minus = some concerns; green plus = low risk; gray circle = not applicable. Abbreviations: ALT4 = alternating trial versus the traditional 4-min condition; ALT8 = alternating trial versus the traditional 8-min condition; APS = agonist–antagonist paired set; BC = biceps curl; BP = bench press; BPull = bench pull; BSQ = back squat; LC = leg curl; LE = leg extension; PAL = paired alternating limb; PS = paired set; RoB 2 = Risk of Bias 2; SQ = squat; SR = seated row; SS = superset; TP = triceps pushdown [9,13,14,16,17,18,19,20,21,42,43,44,45,46,47].

All 50 result-level RoB 2 assessments were judged as having some concerns overall; none were judged at low or high risk overall. Recurring concerns arose mainly from incomplete reporting of the randomization and allocation procedures in Domain 1 and the absence of time-stamped protocols or statistical analysis plans in Domain 5. The clearest outcome-specific difference occurred in Domain 4 (bias in measurement of the outcome). All 18 volume-load results, 20 of 22 completed-repetition results, and nine of 10 training-efficiency results were rated as having “some concerns” in this domain. These judgments reflect the greater reliance of these outcomes on assessor decisions, such as identifying technically valid repetitions or determining momentary muscular failure, while the exercise condition was generally apparent. The scope of the assessments is summarized in Table 3, result-level judgments are presented in Figure 3A–C, and supporting rationales are provided in Supplementary Table S3A–C.

Before reaching consensus, independent ratings from the two reviewers matched the overall RoB 2 judgment in all 50 assessments (100% agreement). Overall Cohen’s κ was not estimable because both reviewers classified every result as having “some concerns”. Across 298 applicable domain-level judgments, exact agreement was 82.9% (247/298) and unweighted Cohen’s κ was 0.67; most disagreements involved Domain 4. Disagreements were resolved through discussion; K.Y.C. was consulted when consensus could not be reached.

#### 3.3.2. Methodological Quality via Modified PEDro

Modified PEDro scores ranged from 5 to 6 out of 7 across the 15 reports. Six reports scored 6/7 and nine scored 5/7. All reports met the modified criteria for randomized allocation or treatment order, retention of more than 85% of participants, analysis according to allocation or treatment order, direct between-condition comparisons, and reporting of point estimates with measures of variability. Allocation concealment was not reported in any report, whereas baseline comparability was demonstrated in 6 of 15 reports. Before consensus, agreement across the seven score-contributing criteria was 91.4% (96/105), with an unweighted Cohen’s κ of 0.72. Criterion-level ratings and modified total scores are presented in Table 4.

### 3.4. Synthesis of Findings by Paired-Set Configuration

Findings are presented for PS-A, PS-B, PS-C_MPR_, and PS-C_EPR_. Each subsection presents the three primary outcomes—volume load, completed repetitions, and training efficiency. Session duration, an intervention-level parameter that varies by protocol design, is reported alongside these outcomes to contextualize the interpretation of training efficiency. Secondary outcomes are then presented separately. Table 5 summarizes the configuration-level descriptive findings for the three primary outcomes. The available estimates are descriptive rather than pooled, and multiple outcomes or comparisons from one underlying study are not independent replications. Because of this non-independence, when at least three underlying studies contributed eligible scalar estimates for a configuration–outcome combination, one representative relative difference per underlying study was used to derive an unweighted study-level median, whereas the full range of available estimates was reported using the lowest and highest scalar estimates across all eligible studies. Importantly, to prevent statistical misinterpretation of this dense synthesis, readers should note that the medians and ranges presented in Table 5 are purely descriptive, unweighted summaries of the individual study estimates; they must not be interpreted as pooled meta-analytic effect sizes or generalizable population effects. For crossover comparisons, 95% confidence intervals were not calculated when paired variability was unavailable; these contrasts are therefore presented as descriptive point estimates. For the parallel-group study by Peña García-Orea et al. [13], review-calculated unstandardized mean differences and 95% confidence intervals are reported. Comparisons involving different TS rest structures were interpreted separately because the magnitude and meaning of a PS-versus-TS contrast depend partly on the recovery provided by the comparator. This dependency was particularly evident in Yamazaki et al. [44], in which the same alternating condition was classified as PS-A when compared with TS using a 4 min rest interval and as PS-B when compared with TS using an 8 min rest interval. Secondary outcomes are presented as reported by the original studies. Because all primary-outcome results had “some concerns” overall on RoB 2, domain-level concerns—particularly the measurement-related concerns for volume load, completed repetitions, and training efficiency—were used to qualify interpretation where relevant.

#### 3.4.1. PS-A: Alternating Exercises with Preserved Rest Intervals

A total of seven PS-A-versus-TS comparisons were identified across six reports representing five underlying studies. In the original inferential analyses, all six comparisons with volume-load data reported statistically significant differences favoring PS-A over TS; however, in Paz et al. [17] and Yamazaki et al. [44], the significant differences were confined to the lat pulldown and squat, respectively. All seven comparisons with completed-repetition data likewise reported statistically significant differences favoring PS-A over TS, although these findings were often exercise- or set-specific and involved the biceps curl, triceps press, leg curl, leg extension, lat pulldown, seated row, bench press, bench pull, and squat. In Robbins et al. [19], the bench-pull advantage was confined to sets 2–3, whereas in de Souza et al. [9], the seated-row and bench-press differences emerged mainly in sets 2–3. Because some comparisons arose from the same report or underlying study, these counts are descriptive and do not represent independent replications; statistical significance was not used to determine the synthesis pattern. Across the available scalar estimates, volume-load estimates ranged from 7.9% to 27.2% above TS, and completed-repetition estimates ranged from 6.3% to 26.7% above TS.

Session-duration estimates were +1.3% in Paz et al. [42], approximately 0% in Robbins et al. [19] and de Souza et al. [9], and 0% in Yamazaki et al. [44]. Reported scalar training efficiency estimates were 3.8% higher in Paz et al. [17] and 21.3–27.2% higher for the bench press and bent-over row in Robbins et al. [19]. Nasser et al. [43] reported significantly higher training efficiency for the agonist–antagonist paired-set and paired alternating upper–lower-limb methods than for TS but did not report exact numerical estimates, whereas de Souza et al. [9] described greater training efficiency without reporting a separate scalar estimate or inferential test. Thus, the training efficiency-related pattern reflected higher work-output estimates within an approximately unchanged session duration. Yamazaki et al. [44] reported lower RPE for lower-body exercise and no statistically detectable difference in upper-body RPE. Among the other PS-A reports that assessed RPE or blood lactate, none reported higher values relative to TS. Two reports additionally provided within-study comparisons between PS-A and PS-C. In de Souza et al. [9], PS-A showed higher later-set repetition values than PS-C, whereas PS-C had the shorter session. In the companion Paz reports, pairwise tests did not detect PS-A-versus-PS-C differences in total volume load or completed repetitions, although paired estimates and confidence intervals for those direct comparisons were unavailable.

#### 3.4.2. PS-B: Alternate Exercise Performed Within the Recovery Period

A total of five PS-B-versus-TS comparisons were identified across five reports representing five underlying studies. Three of the five comparisons reported volume-load data [18,20,44]. In the original inferential analyses, none of these three comparisons detected a statistically significant between-condition difference in volume load. Nevertheless, all available volume-load point estimates were numerically lower under PS-B, ranging from 4.1% to 11.7% below TS. Because paired variability was unavailable for these crossover contrasts, 95% confidence intervals could not be calculated. Accordingly, the nonsignificant findings do not establish equivalence or volume-load preservation, and a possible reduction in volume load under PS-B cannot be excluded. Three of the five comparisons reported completed-repetition data [13,44,45]. Deminice et al. [45] and Yamazaki et al. [44] detected no statistically significant between-condition differences in completed repetitions. In contrast, Peña García-Orea et al. [13] reported significantly fewer total squat repetitions under PS-B, in which the squat and bench press were alternated, whereas the corresponding bench-press total did not differ significantly from TS. Across the available scalar estimates, completed-repetition estimates ranged from 0.7% to 19.5% below TS; the bench-press estimate in Peña García-Orea et al. [13] was 2.1% lower. Based on the reported group-level summary data, the review-calculated mean differences were −17.70 squat repetitions (95% CI, −40.61 to 5.21) and −1.60 bench-press repetitions (95% CI, −21.10 to 17.90). Because these review-calculated intervals were derived from unpaired group-level summary statistics rather than the original study’s within-subject analysis, they are inherently more conservative and should not be interpreted as contradicting the original study’s reported significance.

Session-duration estimates were 44.8–50.0% below TS across all five comparisons. For Peña García-Orea et al. [13], the review-calculated mean difference was −18.90 min (95% CI, −21.57 to −16.23). Deminice et al. [45] reported session durations of 20.2 min under PS-B and 40.3 min under TS, and the two Robbins reports [18,20] completed PS-B sessions in approximately 10 vs. 20 min. Calculable training efficiency estimates from the Robbins reports were 87.8–91.9% higher. In the remaining reports, training efficiency was inferred from protocol timing or described by the authors without a separate inferential analysis; these findings are therefore interpreted primarily as time savings rather than as a common statistically tested training efficiency effect. Neither of the two PS-B reports assessing RPE [44,45] identified an increase relative to TS, and most reported neuromuscular, velocity-based, and metabolic outcomes showed no statistically detectable between-condition differences; Deminice et al. [45] reported higher heart rate. In Yamazaki et al. [44], no statistically significant differences in volume load or completed repetitions were detected between PS-B and TS with 8-min rest. However, the session-duration estimate was 46.8% lower, while the bench-press volume-load and repetition estimates were 11.7% and 12.0% lower and the corresponding squat estimates were 4.2% and 2.9% lower, respectively. Thus, PS-B showed a consistent time-saving pattern, but the available evidence does not establish volume-load equivalence or demonstrate that work output is preserved across all exercises or protocols.

#### 3.4.3. PS-C: Consecutive Paired Exercises Followed by Post-Pair Rest

PS-C produced heterogeneous work-output estimates and shorter session-duration estimates in several protocols. Findings were separated by matched and extended post-pair rest because matched post-pair rest did not imply matched effective recovery. Effective recovery under PS-C also encompassed the time spent performing the intervening exercise and transitioning between exercises.

Matched post-pair rest (PS-C_MPR_). A total of five PS-C_MPR_-versus-TS comparisons were identified across five reports representing five underlying studies [14,16,21,46,47]. All five protocols used post-pair rest matching the 120 s TS interval. Effective recovery before repeating the same exercise was nevertheless longer under PS-C_MPR_; exact end-to-start recovery was available only in Paz et al. [47], at approximately 170 vs. 120 s under TS.

Three of the five comparisons reported volume-load data [16,46,47]. In the original inferential analyses, Bentes et al. [16] reported significantly lower session-total volume load under PS-_CMPR_ than under TS. Paz et al. [46] reported significantly higher seated-row volume load but no statistically significant bench-press difference, whereas Paz et al. [47] reported significantly higher session volume load for both the bench press and seated row. Across the available scalar estimates, volume load ranged from 8.2% below TS in the six-exercise Bentes et al. [16] protocol to 4.5–8.8% above TS in Paz et al. [46] and 11.7–30.1% above TS in Paz et al. [47]. Bentes et al. reported significantly less total work under this protocol, whereas the two single-pair Paz protocols produced exercise-specific or session-level increases. Two comparisons reported completed-repetition data [14,47]. Andersen et al. [14] reported significantly fewer total repetitions per session under PS-C_MPR_, with an estimated 4.0% below TS (218 vs. 227 repetitions). In contrast, Paz et al. [47] reported significantly more seated-row repetitions in sets 1–3 and significantly more bench-press repetitions in sets 2–3. Thus, the original inferential findings and descriptive estimates included both lower and higher work-output results, differing according to exercise and session structure; the Andersen et al. finding reflected a session-total repetition effect, whereas the Paz et al. results were confined to specific exercises and sets.

Three reports provided numerical session-duration data [14,21,47]. Andersen et al. [14] detected a statistically significantly shorter session under PS-C_MPR_; Weakley et al. [21] classified the shorter duration as almost certain using a magnitude-based inference framework, a probabilistic qualitative descriptor distinct from conventional null-hypothesis significance testing; and Paz et al. [47] reported durations of 8.5 vs. 16.0 min without a separate inferential test of session duration. Across these reports, session-duration estimates were 39.7–46.9% below TS. Weakley et al. [21] was the only report to provide a scalar training efficiency estimate, which was 75.7% higher than TS and was classified as almost certainly greater under the study’s magnitude-based framework. Other reports described favorable training efficiency findings without conducting a separate inferential analysis. Both reports assessing RPE found higher values under PS-C_MPR_: Andersen et al. [14] reported a statistically significant increase, whereas Weakley et al. [21] classified the increase as almost certain using magnitude-based inference. Paz et al. [47] also reported significantly greater EMG fatigue indices under PS-C_MPR_. Weakley et al. [21] reported very likely to almost certainly greater blood lactate responses and a very likely greater increase in creatine kinase at 24 h. These protocols therefore showed consistently shorter session-duration estimates in the available data, whereas volume-load and completed-repetition findings varied by exercise and session scale and were accompanied by higher fatigue-related responses in the reports that assessed them.

Regarding extended post-pair rest (PS-C_EPR_), three reports, representing two underlying studies, contributed two PS-C_EPR_-versus-TS comparisons [9,17,42]: one from de Souza et al. [9] and one from the companion Paz reports [17,42], which were treated as a single underlying study but cited separately according to the outcomes each article reported, rather than as independent replications. Only Paz et al. [17] provided a usable scalar volume-load estimate for PS-C_EPR_. In the original inferential analysis, session volume load was significantly higher than TS, with a descriptive estimate 9.6% above TS. De Souza et al. [9] did not provide a scalar volume-load estimate suitable for the present synthesis. Both underlying-study comparisons reported completed-repetition findings. Paz et al. [17] reported significantly more total repetitions per session under PS-C_EPR_, with an estimate 8.2% above TS. In contrast, de Souza et al. [9] detected no statistically significant repetition differences between PS-C_EPR_ and TS; the seated-row set differences were 0.0, −0.6, and −0.6 repetitions, and the bench-press differences were −0.1, −0.3, and −0.7. The available results therefore do not support one common completed-repetition pattern across extended-post-pair-rest protocols.

Time-related estimates also differed. De Souza et al. [9] reported session durations of approximately 5 min under PS-C_EPR_ and 10 min under TS (a 50% reduction), but they did not report a separate inferential test of session duration. In Paz et al. [42], no statistically significant protocol effect on session duration was detected; nevertheless, the PS-C_EPR_ point estimate was 4.4% above TS. The protocol structures also differed. De Souza et al. used no rest between the paired exercises followed by 150 s of post-pair rest, whereas TS used 120 s intervals; the Paz protocol used 180 s of post-pair rest compared with 90 s under TS. In Paz et al. [17], the training efficiency estimate was 0.8% above TS, but the original analysis detected no statistically significant protocol effect on training efficiency. De Souza et al. [9] described the PS-C_EPR_ condition as more time-efficient but did not report a separate scalar estimate or inferential test of training efficiency. Accordingly, these results do not establish either equivalence or one common training efficiency effect across the two extended-post-pair-rest protocols. De Souza et al. [9] reported significantly higher RPE and blood lactate under PS-C_EPR_, whereas the companion Paz reports [17,42] did not identify statistically detectable increases in RPE or the reported muscle damage and metabolic markers. Pairwise tests in the companion Paz reports [17,42] did not detect PS-A-versus-PS-C_EPR_ differences in total volume load or completed repetitions, although paired estimates and confidence intervals for those direct comparisons were unavailable; because both reports arose from the same underlying study, these findings represent within-study protocol variation rather than independent replication across configurations.

## 4. Discussion

This systematic review evaluated the acute mechanical and perceptual effects of distinct paired-set configurations versus traditional sets. Overall, recovery distribution appeared to be an important determinant of performance patterns: PS-A, characterized by longer rest intervals between identical exercises, maintained or elevated volume load and training efficiency while preserving repetition performance in an exercise-specific manner. PS-B, which interspersed an alternative exercise during the rest interval, achieved substantial gains in training efficiency, although its capacity to preserve volume load and repetitions remained inconclusive. In contrast, PS-C_MPR_, involving near-zero rest between paired exercises with post-pair rest comparable to traditional sets, maximized training efficiency but elevated perceived exertion and produced heterogeneous performance outcomes. Finally, based on limited evidence, PS-C_EPR_, featuring longer rest intervals between exercises targeting different muscle groups, demonstrated protocol-specific trade-offs between work output and session duration.

### 4.1. PS-A: A Work-Output Pathway—Consistency Across PS-A Protocols

PS-A showed higher work-output estimates across markedly different protocols than TS, with volume-load estimates 7.9–27.2% and completed-repetition estimates 6.3–26.7% above TS where scalar data were available. The PS-A evidence base spanned three sets at 4RM in Robbins et al. [19], three sets at 8RM in de Souza et al. [9], three sets at 10RM in the companion Paz reports, three sets at 15RM in Nasser et al. [43], and five sets at 85% 1RM to momentary failure in Yamazaki et al. [44]. It also included both agonist–antagonist and upper–lower pairings, indicating that the favorable work-output pattern was not confined to one load, repetition range, set number, or pairing type. These estimates were not pooled and should therefore be interpreted as recurring study-level point estimates rather than a common effect size. The clearest common protocol feature was that PS-A preserved the comparator’s full rest interval after each exercise and thereby lengthened recovery before the same exercise was repeated. This provides a plausible explanation for why higher work-output point estimates could have occurred with approximately unchanged session duration.

Evidence from the 14 included studies and 15 reports indicated that while PS-A generally demonstrated better repetition performance than TS, this advantage was exercise-specific rather than consistent across all movements. As summarized in Table 2, repetition improvements were identified in exercises such as the biceps curl, triceps press, leg extension, lat pulldown, bench pull, bench press, seated row, and squat; however, individual studies showed significant effects only in select movements (e.g., lat pulldown in Paz et al. [17] vs. squat in Yamazaki et al. [44]). One plausible explanation is that PS-A preserved the rest interval used after each exercise in its TS comparator while alternating between two movements, thereby extending the recovery period before the same exercise was performed again. Even under comparable session durations, this recovery structure may have delayed the accumulation of localized neuromuscular and metabolic fatigue, potentially helping to preserve repetition performance [48]. This was consistent with evidence showing that agonist–antagonist configurations sustained volume load within equivalent or reduced durations, whereas insufficient rest may compromise work capacity, suggesting that rest distribution may contribute importantly to performance [10]. Additionally, the advantage of PS-A typically emerged in later sets (sets 2–3 in Robbins et al. [19] and de Souza et al. [9]) rather than the initial set. Given that volume load still exhibited a set-by-set decline, PS-A did not abolish fatigue, but rather delayed its accumulation across the session. These explanations remain tentative because the included studies were not designed to isolate the mechanisms.

### 4.2. PS-B: A Time-Compression Pathway—Time Savings with Variable Work Output

The clearest advantage of PS-B was shorter session duration because the alternate exercise replaced time that would otherwise have been passive recovery. Across the five comparisons, session-duration estimates were 44.8–50.0% below TS. The two Robbins reports completed paired protocols in approximately 10 rather than 20 min, while Deminice et al. [45] reduced duration from 40.3 to 20.2 min. This time-compression effect is consistent with previous findings [22,23]. Importantly, although Table 2 indicates that three of the five studies reporting volume load found no statistically significant differences between PS-B and TS, descriptive point estimates showed that PS-B yielded 4.1–11.7% lower volume load and 0.7–19.5% fewer completed repetitions relative to TS. Therefore, current evidence only confirms that PS-B serves as an effective time-compression strategy; whether it truly preserves volume load and repetition capacity remains inconclusive and warrants further verification in future studies. Notably, Peña García-Orea et al. [13] observed a reduction in squat repetitions under PS-B, which was likely to be attributable to their set-termination criteria using a 15% velocity loss threshold for the back squat and 20% for the bench press. Velocity loss represents a validated indicator of fatigue; as neuromuscular fatigue accumulates, barbell velocity declines, and a more stringent cutoff threshold terminates sets after fewer completed repetitions [49,50], thereby forcing earlier set cessation in the squat. However, the squat result may therefore reflect the velocity-loss termination rule, but these possibilities were not tested independently. Furthermore, the previous literature has demonstrated that multi-joint lower-body exercises impose greater systemic recovery demands [51,52]; interleaving bench press sets between successive squat bouts reduced inter-set recovery, which is likely to have compromised squat performance. Ycamazaki et al. [44] further illustrates why comparator recovery structure matters. compared with a TS protocol with an 8 min rest interval (classified as a PS-B comparison), the paired-set configuration shortened session duration from approximately 77 to 41 min; however, bench-press and squat volume-load point estimates were approximately 12% and 3–4% lower, respectively, although the reductions in volume load and completed repetitions did not reach statistical significance. Conversely, compared with a TS condition with 4 min rest intervals (classified as PS-A comparison), the same alternating sequence effectively doubled same-exercise recovery duration, thereby producing higher squat work output without altering session duration. The interpretation of an alternating sequence therefore depends not only on exercise order but also on how recovery is distributed relative to the comparator.

### 4.3. PS-C: A Rest-Adaptive Pathway—Work Retention with Variable Volume-Load Efficiency

We categorized PS-C_MPR_ and PS-C_EPR_ according to whether post-pair rest matched or exceeded the corresponding TS rest interval. PS-C_MPR_ showed heterogeneous work-output estimates that appeared to vary with session scale. Across the included studies, the acute effects of PS-C_MPR_ on volume load and repetition performance exhibited pronounced heterogeneity, which may have been largely moderated by pair number, targeted muscle groups, and overall session volume. In single-pair upper-body protocols involving two exercises across three sets, PS-C_MPR_ consistently maintained or significantly enhanced VL output (e.g., seated row in Paz et al. [46]; all exercises in Paz et al. [47]), with point estimates indicating increases ranging from 4.5% to 30.1%. Conversely, when protocols expanded to multi-pair or whole-body configurations involving six to eight exercises, three to four pairs, and high total set volumes, lower work-output estimates were observed in some studies [14,16], including an 8.2% lower volume-load estimate in Bentes et al. [16] and approximately 4.0% fewer completed repetitions in Andersen et al. [14]. This divergence indicated that when paired configurations were applied across extensive full-body sessions, the compressed intra-pair recovery progressively compounded metabolic and localized muscular fatigue, impairing repetition retention in later exercises. One plausible physiological explanation is that restricted phosphocreatine (PCr) resynthesis and accelerated anaerobic glycolysis may contribute to the accumulation of lactate, hydrogen ions (H+), and related metabolites, which could disrupt excitation–contraction coupling and attenuate contractile force [53]. Moreover, Mang et al. [23] also noted that fatigue may accumulate as more exercises and sets are added. However, these mechanisms remain hypothesized explanations derived from previous literature rather than mechanisms directly demonstrated by the included studies. Despite modest repetition declines in larger multi-pair protocols, PS-C_MPR_ demonstrated a consistent training-efficiency advantage. In Andersen et al. [14], an approximate 4.2% reduction in completed repetitions was offset by a ~40% reduction in session duration (from 58 to 35 min), resulting in higher volume-load efficiency than TS. However, higher volume load completed per minute does not inherently translate into superior chronic muscular adaptations and fails to capture essential determinants of training quality, including movement execution, technical competency, acute endocrine or neuromuscular stress, and cumulative fatigue. In the present synthesis, two investigations reported significantly higher ratings of perceived exertion (RPE) under PS-C_MPR_ than TS. This finding is plausibly explained by the high-volume protocols employed by Andersen et al. [14] and Weakley et al. [21] (18–24 total sets), in which condensed work density without passive intra-pair rest may have increased subjective fatigue. Accordingly, fatigue responses may depend on session scale, exercise selection, and total prescribed volume. Practitioners should therefore interpret high volume-load efficiency in conjunction with movement competency, execution quality, and fatigue status, rather than merely quantifying external load, completed repetitions, and elapsed training time.

The evidence for paired sets with PS-C_EPR_ was limited, stemming from only two independent studies (three reports total). The two Paz reports [17,42] were treated as companion reports from one underlying study and were used for the outcomes reported in each article rather than counted as independent replications. Among these protocols, only the Paz companion study, which used extended post-pair rest relative to TS, showed higher volume load and more repetitions under PS-C_EPR_ [17,42]. Across the two PS-C_EPR_ protocols, training-efficiency and RPE responses differed, alongside differences in session duration and rest structure; these differences may therefore help explain the contrasting volume-load and repetition responses [9,17,42]. When the total session duration of PS-C_EPR_ was very close to that of TS, both VL and repetitions increased; for instance, Paz et al. [17] showed that the estimated VL for PS-C_EPR_ was 9.6% higher and completed repetitions were 8.2% higher. Conversely, when total session duration was reduced by roughly 50%, repetitions showed no significant difference from TS [9]. Additionally, training efficiency yielded inconsistent results across protocols. In de Souza et al. [9], session duration was reduced from approximately 10 to 5 min, and the authors described the PS-C_EPR_ condition as more time-efficient; however, no separate inferential test of training efficiency was reported. In Paz et al. [17], however, despite an increase in repetitions, training efficiency was only 0.8% higher than TS, which was not statistically significant. A possible reason for this lack of significance was that extending the post-pair rest increased the overall session time by 4.4%, potentially diluting the time-saving efficiency gains; furthermore, the large standard deviation (SD) in the VL data could also have been a contributing factor to the non-significant result. Finally, session duration and work density may have influenced RPE. De Souza et al. [9] employed a 150 s post-set rest (TS was 120 s), and with differences in repetitions ranging only from 0.0 to −0.7 across sets, compressed the total session duration from about 10 min to 5 min. This higher work density may have contributed to earlier fatigue and was accompanied by higher blood lactate and RPE. A previous study indicated that compared to traditional sets, paired-set training resulted in 18.3% significantly higher blood lactate levels, 7.8% higher average heart rate, and significantly higher RPE [54]. This increase in metabolic stress is hypothesized to be related to the perception of effort, as higher blood lactate levels may potentially exacerbate discomfort and perceived exertion [54]. However, it should be noted that this mechanistic chain was primarily an inference based on previous findings; while published evidence supported the link between high-density training, metabolic stress, and elevated RPE, this complete causal pathway was not directly verified within the two studies included in the present review.

### 4.4. Strengths and Limitations

The present study is the first to systematically deconstruct distinct pairing configurations—specifically, the inter-set rest interval within paired exercises, the rest interval following paired exercises, and the rest interval between successive sets of the same exercise—to investigate their effects on training volume load, repetition performance, and training efficiency, thereby offering potential practical applications for resistance training prescription. Nevertheless, the present review incorporated 15 reports encompassing 14 original studies with a total of 223 participants (208 males, 15 females; notably, Andersen et al. [14] was the sole report to include female participants). Thirteen of the included studies employed a randomized crossover design, while one utilized a randomized parallel-group design. The limitations of this review are delineated across the following dimensions.

Regarding the data-related limitations of the included literature, first, the generalizability of participant characteristics is constrained. The sample size of each included study was relatively small, with a mean participant age ranging from 20 to 28 years, and all participants possessed prior resistance training experience. Consequently, the findings should not be directly extrapolated to females, older adults, or untrained populations. Second, the included studies exclusively evaluated acute resistance-exercise responses, thereby precluding the establishment or extrapolation of longitudinal training adaptations. Third, the paucity of literature concerning lower-body-dominant training protocols restricts the interpretation and application of acute lower-body training responses. Fourth, the insufficient number of independent studies per primary outcome across configurations compromises the representativeness of the data; this sparsity precludes a meaningful quantitative assessment of publication bias and cannot definitively rule out the presence of publication bias.

Regarding methodological limitations, first, this review was not prospectively registered and lacked a formal protocol. Although the PICOS inclusion criteria and primary outcomes were established prior to literature screening and the review was conducted in accordance with the planned procedures, the risks of selective reporting and post hoc methodological adjustments cannot be entirely eliminated. The PS-A, PS-B, PS-C_MPR_, and PS-C_EPR_ labels were developed after data extraction as review-specific organizational categories and should not be interpreted as an established taxonomy in the broader resistance-training literature. Second, restricting eligibility to English-language reports may have excluded relevant evidence published in other languages. Third, regarding the risk-of-bias appraisal, all 50 result-level assessments of the three primary outcomes using the RoB 2 tool were rated as having “some concerns” overall, with measurement-related concerns in Domain 4 commonly identified with regard to volume load, completed repetitions, and training efficiency. Crucially, the practical meaning of this consistent appraisal is twofold. On one hand, this rating does not imply that all included studies are equally limited in scientific rigor; many of these studies demonstrated exceptional experimental control over training volume and execution, yet were consistently categorized under “some concerns” due to unavoidable, field-wide methodological challenges reflected in Domain 4, such as the inherent impossibility of blinding participants and investigators during active training, and the historical lack of prospective trial registration. On the other hand, these recurring measurement-related uncertainties indicate that, although the observed acute performance and training efficiency trends are highly informative, they should be regarded as preliminary indications rather than as definitive, generalizable conclusions. Coaches and practitioners are therefore encouraged to apply these findings cautiously and on an individualized basis. Thus, this uniform rating serves as a call for cautious, individualized application in practice rather than a dismissal of the studies’ scientificity. In addition to the Cochrane risk-of-bias assessment, this study employed a modified PEDro scale, with the total score adjusted to 7 after removing the three blinding items that are inapplicable to exercise testing. The included studies scored between 5 and 6, presenting a risk of study-quality overestimation that cannot be ruled out. Notably, all studies failed to report allocation concealment, and only six reports demonstrated baseline comparability, thereby limiting confidence in baseline similarity and the subsequent interpretation of the measured outcomes.

Finally, because most of the included studies used crossover designs, the absence of paired standard deviations or within-participant correlation coefficients precluded the calculation of 95% confidence intervals for most crossover mean differences, which in turn limited the ability to quantitatively synthesize the evidence. Together with heterogeneity in training protocols and outcome measures and the presence of dependent comparisons, these limitations did not support an interpretable pooled estimate. A meta-analysis was therefore not conducted; instead, available quantitative data were summarized descriptively rather than statistically pooled. This review accordingly relied on a descriptive synthesis incorporating reported condition means, unstandardized mean differences, and descriptive relative differences where available, which constitutes a methodological limitation that may weaken the overall strength of the empirical evidence.

These limitations highlight key priorities for future research across four domains: sample diversity, exercise selection, intervention duration, and physiological mechanisms. To bolster generalizability, future trials should enroll females, older adults, and untrained populations. Additionally, implementing lower-body-dominant protocols is essential to resolve current evidence asymmetries between upper- and lower-limb musculature. Methodologically, research must progress from acute, single-session designs to longitudinal training studies. In their review, Cowley et al. [55] identified only three long-term investigations on paired-set training, highlighting a scarcity of evidence on chronic adaptations. Prospective longitudinal trials are therefore warranted to verify chronic adaptations in strength, hypertrophy, and endurance. Furthermore, comprehensive mechanistic evaluations incorporating muscle oxygenation, phosphocreatine resynthesis, and central fatigue are necessary to elucidate the physiological underpinnings of paired-set performance. Finally, to improve reproducibility and facilitate quantitative synthesis, it is essential that future reports explicitly delineate timing boundaries and systematically report intra- and post-pair rest intervals, effective same-exercise recovery durations, mechanical work, total session duration, and multi-dimensional fatigue metrics.

## 5. Conclusions

Based on the available acute-response evidence in young resistance-trained adults, paired-set resistance training should be prescribed according to specific session objectives. PS-A provided the most consistent evidence for higher volume-load and completed-repetition estimates without substantially extending session duration. In time-constrained scenarios, PS-B consistently shortened training time, although mechanical work output may not be preserved, particularly during demanding lower-body exercises. While constrained by a limited evidence base, PS-C configurations may afford greater programming flexibility; however, its work-retention and time-saving profiles remain highly contingent upon post-pair rest intervals, exercise selection, and total session scale. In addition to these configuration-specific limitations, these findings are derived from acute designs, and all evaluated studies exhibited “some concerns” according to the RoB 2 tool; accordingly, these conclusions should be interpreted with appropriate caution when translated into practice. Ultimately, coaches and strength and conditioning practitioners should collectively balance mechanical work, available session time, and perceived exertion when selecting an optimal paired-set configuration.

## Figures and Tables

**Table 1:** PICOS Eligibility Criteria.

PICOS Component	Inclusion Criteria	Exclusion Criteria
Population	Healthy, resistance-trained adults aged ≥18 years, regardless of sex; ≥6 months of continuous resistance-training experience; ≥1 resistance-training session per week; training status consistent with at least Tier 2 (trained/developmental) where sufficient information was available.	Untrained participants; participants aged <18 years; populations with chronic disease, musculoskeletal disorders, or active/acute injuries that did not meet the review population criteria.
Intervention	Single-session dynamic resistance exercise using a PS configuration of two different exercises targeting different primary muscle groups (e.g., agonist–antagonist, upper–lower, or otherwise unrelated muscle groups); ≥3 sets performed for each exercise.	Static or isometric protocols; single-exercise protocols; same-primary-muscle pairings (e.g., pre-exhaustion or compound sets); tri-sets or circuits involving ≥3 exercises within the repeated sequence; <3 sets performed per exercise.
Comparator	TS configuration using the same exercises, number of sets, and prescribed loads or relative intensities as the PScondition.	Comparators using different exercises, numbers of sets, loads, or relative intensities from the PS condition.
Outcomes	Acute volume load, completed repetitions, and/or training efficiency; secondary outcomes extracted when reported to provide context for the primary outcomes.	Studies without usable data for at least one prespecified primary outcome.
Study design	Randomized single-session acute trials, including randomized crossover and randomized parallel-group designs.	Chronic longitudinal training interventions; nonrandomized trials; case studies; reviews; conference abstracts; non-peer-reviewed reports.

Note. PICOS = Population, Intervention, Comparator, Outcomes, and Study Design; PS = paired set; TS = traditional set.

**Table 2:** Study and Protocol Characteristics and Outcome-Specific Findings of the Included Reports.

Study Report	Design	Participants [*n*, Sex, Age (yr)]	Trial (A/B/C/TS)	Same-Exercise Recovery [Ex_1_ → Next Ex_1_ (s)]	Session Duration (min)	Exercise Pairing Order (Type; Ex_1_ → Ex_2_)	Exercise Protocol (ex; Sets × Intensity)	Comparison (X vs. Y)	Primary Outcomes			Secondary Outcomes
									VL (kg)	Reps (Count)	EFF (kg·min^−1^)	
Nasser et al. (2019) [43]	RCO	12 male 28.1 ± 4.8	TS	60	20		4 ex; 3 × 15RM					
			A	120+	20	ag–an: BC → TP; LC → LE		APS vs. TS	↑	↑ (BC, TP & LE)	↑	RPE—
			A	120+	20	up–low: BC → LC; TP → LE		PAL vs. TS	↑	↑ (BC, LC & LE)	↑	RPE—
								APS vs. PAL	—	—	—	RPE—
Paz et al. (2019a) [42]	RCO	13 male 26.2 ± 3.9	TS	90	39.0 ± 2.8		6 ex; 3 × 10RM					
			A	180+	39.5 ± 3.4	ag–an: LBP → LPD; BP45 → SCR; TE → BC		PS-A vs. TS	↑	↑		RPE—
			C_EPR_	180+	40.7 ± 2.0	ag–an: LBP → LPD; BP45 → SCR; TE → BC		PS-C vs. TS	↑	↑		RPE—
								PS-A vs. PS-C	—	—		RPE—
Paz et al. (2019b) [17]	RCO	22 male 25.2 ± 4.1	TS	90	≈45 ± 3.5		6 ex; 3 × 10RM					
			A	180+	≈45 ± 3.5	ag–an: LBP → LPD; BP45 → SCR; TE → BC		PS-A vs. TS	↑ (LPD)	↑ (LPD)	—	RPE & LAC— EMG (SCR–LD) ↓
			C_EPR_	180+	≈45 ± 3.5	ag–an: LBP → LPD; BP45 → SCR; TE → BC		PS-C vs. TS	↑	↑	—	RPE & LAC— EMG (BC–LD) ↓
								PS-A vs. PS-C	—	—	—	LAC—
Robbins et al. (2010b) [19]	RCO	16 male 23.6 ± 4.7	TS	120 ‡	≈10 ‡		2 ex; 3 × 4RM					
			A	≈240 ‡	≈10 ‡	ag–an: Bpull → BPr		PS-A vs. TS	↑	↑ (Bpull: S2–3; Bpress: S1–3)	↑ †	
Yamazaki et al. (2025) [44]	RCO	13 male 22.3 ± 3.9	TS-4 min	240	41 ± 1		2 ex; 5 × 85% 1RM					
			TS-8 min	480	77 ± 1							
			A	480+	41 ± 1	up–low: BPr → SQ		PS-A vs. TS-4	↑ (SQ)	↑ (SQ)		RPE (LB) ↓
			B	480+	41 ± 1	up–low: BPr → SQ		PS-B vs. TS-8	—	—	↑ †	RPE—
de Souza et al. (2017) [9]	RCO	10 male 27.5 ± 3.8	TS	120	≈10		2 ex; 3 × 8RM	PS-A vs. TS		↑ (S2–3; SR & BPr)	↑ †	RPE & LAC—, FI ↑
			A	240+	≈10	ag–an: SR → BPr		PS-C vs. TS		—	↑ †	RPE (S2–3) & LAC ↑, FI ↓
			C_EPR_	150+	≈5	ag–an: SR → BPr		PS-A vs. PS-C		↑ (S2–3; SR & BPr)		RPE (S2–3) & LAC ↓, FI ↑
Robbins et al. (2010a) [18]	RCO	18 male 24.6 ± 5.1	TS	240 ‡	≈20 ‡		2 ex; 3 × 4RM + 3 × 4 @ 40% 1RM					
			B	≈240 ‡	≈10 ‡	ag–an: Bpull → BPT		PS-B vs. TS	—		↑ †	EMG—
Robbins et al. (2010c) [20]	RCO	16 male 23.8 ± 5.2	TS	240 ‡	≈20 ‡		2 ex; 3 × 4RM					
			B	≈240 ‡	≈10 ‡	ag–an: Bpull → BPr		PS-B vs. TS	—		↑ †	EMG—
Deminice et al. (2011) [45]	RCO	11 male 25.9 ± 2.8	TS	90	40.3 ± 1.8		6 ex; 3 × 75% 1RM					
			B	90	20.2 ± 1.6	up–low: BPr → LE; CPD → LF; OHP → LP		PS-B vs. TS		—	↑ †	RPE & LAC—
Peña García-Orea et al. (2022) [13]	RPG	19 male 24.0 ± 5.0	TS	180	42.2 ± 3.1		2 ex; 3 × 55–70% 1RM (VLoss: 15% SQ; 20% BPr)					
			B	180	23.3 ± 2.2	up–low: SQ → BPr		PS-B vs. TS		↓ (SQ total)	↑ †	
Bentes et al. (2017) [16]	RCO	13 male 20.0 ± 1.3	TS	120	NR		6 ex; 3 × 10RM					
			C_MPR_	120+	NR	ag–an: CP → LR; LE → LC; PD → SP		PS-C vs. TS	↓			
Paz et al. (2014) [46]	RCO	15 male 22.2 ± 2.3	TS	120	NR		2 ex; 3 × 8RM					
			C_MPR_	120+	NR	ag–an: BPr → SR		PS-C vs. TS	↑ (SR)			
Paz et al. (2017) [47]	RCO	15 male 22.4 ± 1.1	TS	120	16.0 ± 3.5		2 ex; 3 × 10RM					
			C_MPR_	≈170	8.5 ± 2.3	ag–an: BPr → SR		PS-C vs. TS	↑	↑ (SR: S1–3; BPr: S2–3)	↑ †	EMG fatigue indices ↑ (LD & PM: S1–3; BB & TB: S2–3) ↑
Andersen et al. (2022) [14]	RCO	29 (15 female, 14 male) 27.2 ± 7.2	TS	120	58 ± 3		8 ex; 3 × ≈9RM					
			C_MPR_	120+	35 ± 3	mixed: DL → BPr; SQ → SealR; Fly → TE; RF → BC		PS-C vs. TS		↓	↑ †	RPE ↑
Weakley et al. (2017) [21]	RCO	14 male 20.8 ± 1.2	TS	120	42.3 ± 1.3		6 ex; 3 × 65% 3RM					
			C_MPR_	120+	24.0 ± 1.2	mixed: SQ → BPr; RDL → SP; BOR → UR		PS-C vs. TS			↑ ᵐ	RPE & LAC ↑ ᵐ

Note. Comparisons are X versus Y. In outcome columns, ↑ and ↓ indicate a source-supported higher or lower result in X; — indicates that no between-protocol difference was reported. Unless marked † or ᵐ, symbols are supported by a direct inferential comparison. Blank cells indicate that the outcome was not reported or that no usable direct comparison was available. TS rows provide comparator protocol details only. Values are mean ± SD unless otherwise stated; session duration is reported in minutes and rest intervals in seconds. Exercise pairing order is shown as Ex_1_ → Ex_2_; multiple pairs are separated by semicolons. † Author-reported directional or comparable result without a direct inferential comparison of that outcome. For EFF, † denotes the original authors’ explicit description of training efficiency; no efficiency value was calculated by the review authors. ᵐ Author-reported magnitude-based inference. + The displayed same-exercise value includes only protocol-specified rest or transition intervals; actual recovery also includes the intervening Ex_2_ set. ≈ Source-reported approximate value or a value containing a source-reported approximate transition. ‡ Robbins et al. (2010a–c) [18,19,20] reported nominal timing anchored to set beginnings or midpoints; the displayed intervals should not be summed as exact passive rest. Trial labels A, B, C_MPR_ and C_EPR_ correspond to paired-set configurations. PS-A retains the comparator’s full rest interval after each exercise; PS-B inserts the paired exercise within the comparator’s same-exercise recovery window; PS-C_MPR_ executes paired exercises consecutively followed by a post-pair rest interval matching TS; and PS-C_EPR_ executes paired exercises consecutively followed by an extended post-pair rest interval exceeding TS, as operationalized in the paired-set taxonomy. Classification was review-defined, descriptive, and applied at the comparison level. Abbreviations: ag–an = agonist–antagonist; APS = agonist–antagonist paired set; EFF = training efficiency; ex = exercises; C_EPR_ = extended post-pair rest; min = minutes; mixed = more than one pairing relation; C_MPR_ = matched post-pair rest; *n* = sample size; NR = not reported; PAL = paired alternating upper–lower-limb method; PS = paired set; RCO = randomized crossover design; reps = repetitions; RM = repetition maximum; RPG = randomized parallel-group design; s = seconds; S = set; SD = standard deviation; TS = traditional set; up–low = upper–lower body; VL = volume load; yr = years. Exercise and outcome codes: BB = biceps brachii; BC = biceps curl; BOR = bent-over row; BP45 = 45° incline bench press; BPr = bench press; Bpress = bench press; BPT = bench-press throw; Bpull = bench pull; CP = chest press; CPD = cable pulldown; DL = deadlift; EMG = electromyography; FI = fatigue index calculated from repetition performance [(third-set repetitions/first-set repetitions) × 100; higher values indicate less fatigue]; Fly = chest fly; LAC = blood lactate; LB = lower body; LBP = lying bench press; LC = leg curl; LD = latissimus dorsi; LE = leg extension; LF = leg flexion; LP = leg press; LPD = lat pulldown; LR = low row; OHP = overhead press; PD = pulldown; PM = pectoralis major; RDL = Romanian deadlift; RF = reverse fly; RPE = rating of perceived exertion; SCR = seated close-grip row; SealR = seal row; SP = shoulder press; SQ = squat/full squat; SR = seated or wide-grip row; TB = triceps brachii; TE = triceps extension; TP = triceps press; UR = upright row; VLoss = velocity loss.

**Table 3:** Scope of RoB 2 Assessments by Primary Outcome and Paired-Set Configuration.

Outcome	Assessments, (*n*)	Study Reports, (*n*)	Underlying Studies, (*n*)	PS-A, (*n*)	PS-B, (*n*)	PS-C, (*n*)
Volume load	18	9	9	7	5	6
Completed repetitions	22	7	7	13	5	4
Training efficiency	10	6	6	5	3	2
Overall	50	14	14	25	13	12

Note. RoB 2 assessments were restricted to the three primary outcomes: volume load, completed repetitions, and training efficiency. Session duration was recorded as an intervention-level parameter rather than an outcome and was therefore not included in the RoB 2 assessment. Study-report and underlying-study counts are unique within each outcome; because reports and studies could contribute to more than one outcome, counts across outcome rows are not additive. PS-A, PS-B, and PS-C were classified according to the definitions provided earlier in the manuscript. The PS-A–PS-C columns count result-level assessments, not independent studies. RoB 2 assessments of training efficiency were limited to identifiable efficiency estimates or analyses. Author-reported descriptive training efficiency statements marked with † in Table 2 were retained in the synthesis but were not treated as separate RoB 2 results. PS = paired set; RoB 2 = Risk of Bias 2.

**Table 4:** Modified PEDro Ratings of the 15 Included Study Reports.

Study Report	1	2	3	4	8	9	10	11	Total (/7)
Andersen et al. (2022) [14]	1	1	0	0	1	1	1	1	5
Bentes et al. (2017) [16]	0	1	0	1	1	1	1	1	6
Peña García-Orea et al. (2022) [13]	0	1	0	0	1	1	1	1	5
Paz et al. (2014) [46]	0	1	0	0	1	1	1	1	5
Paz et al. (2017) [47]	0	1	0	0	1	1	1	1	5
Paz et al. (2019a) [42]	0	1	0	1	1	1	1	1	6
Paz et al. (2019b) [17]	0	1	0	1	1	1	1	1	6
Robbins et al. (2010a) [18]	1	1	0	0	1	1	1	1	5
Robbins et al. (2010b) [19]	1	1	0	0	1	1	1	1	5
Robbins et al. (2010c) [20]	1	1	0	0	1	1	1	1	5
Weakley et al. (2017) [21]	1	1	0	1	1	1	1	1	6
Nasser et al. (2019) [43]	0	1	0	0	1	1	1	1	5
Deminice et al. (2011) [45]	1	1	0	1	1	1	1	1	6
de Souza et al. (2017) [9]	0	1	0	1	1	1	1	1	6
Yamazaki et al. (2025) [44]	0	1	0	0	1	1	1	1	5

Note. PEDro = Physiotherapy Evidence Database. A rating of 1 indicates that the criterion was explicitly satisfied; 0 indicates that the criterion was not satisfied or was insufficiently reported. Item 1 = eligibility criteria and source of participants; item 2 = random allocation or randomized condition order; item 3 = allocation concealment; item 4 = baseline similarity; item 8 = outcome data available for more than 85% of participants; item 9 = analysis according to the allocated condition or intention-to-treat; item 10 = between-condition statistical comparisons; item 11 = point estimates and measures of variability. Item 1 was retained for descriptive purposes but was not included in the total score. Items 5–7, concerning blinding of participants, intervention providers, and outcome assessors, were excluded under the modified PEDro approach. The modified total is the sum of items 2–4 and 8–11 (maximum = 7).

**Table 5:** Descriptive Synthesis of Acute Outcome Patterns by Paired-Set Configuration.

Configuration and Rest Structure	Evidence Base	Volume Load	Completed Repetitions	Training Efficiency	Session Duration
PS-A Alternating configuration Full TS rest was retained after each exercise; recovery before repeating the same exercise was longer than TS.	6 reports 7 comparisons 5 underlying studies	Median: +16.5% (4 studies) Range: +7.9% to +27.2%	Median: +15.0% (3 studies [17,43,44]) Range: +6.3% to +26.7% 1 study [9]: differences occurred mainly in sets 2–3.	Available estimates: +3.8% to +27.2% (2 studies [17,19]). 2 studies: descriptive advantage only.	Median: 0.0% (4 studies) Range: 0.0% to +1.3%
PS-B Rest-embedded configuration The alternate exercise was performed during recovery before the same exercise was repeated.	5 reports 5 comparisons 5 underlying studies	Median: −5.4% (3 studies) Range: −11.7% to −4.1%	Median: −7.4% (3 studies) Range: −19.5% to −0.7%	Available estimates: +87.8% to +91.9% (2 studies). Other reports primarily supported time savings.	Median: −49.9% (5 studies) Range: −50.0% to −44.8%
PS-C_MPR_ Matched post-pair rest Post-pair rest matched TS; effective recovery also included the intervening exercise.	5 reports 5 comparisons 5 underlying studies	Median: +6.7% (3 studies) Range: −8.2% to +30.1%	Andersen: session-total estimate 4.0% below TS. Paz et al. (2017) [47]: higher in seated-row sets 1–3 and bench-press sets 2–3.	Weakley: 75.7% higher volume-load-per-minute estimate. Other timed reports described favorable efficiency.	Median: −43.3% (3 studies) Range: −46.9% to −39.7%
PS-C_EPR_ Extended post-pair rest Post-pair rest exceeded TS; effective recovery further included the intervening exercise.	3 reports 2 comparisons 2 underlying studies	Paz companion report: session-total estimate +9.6% vs. TS. ^a^ No scalar estimate was available in de Souza.	Paz: session-total estimate +8.2%. ^a^ de Souza: set-level differences ranged from 0.0 to 0.7 fewer repetitions under PS-C than TS.	Paz: estimate +0.8%. ^a^ de Souza: greater efficiency described without a scalar estimate.	de Souza: approximately −50.0%. Paz: estimate +4.4%. ^b^

Note. Percent differences were calculated from reported condition means as (PS − TS)/TS × 100. Medians are unweighted study-level summaries and were reported only when at least three underlying studies provided comparable scalar estimates. Ranges show the lowest and highest available scalar estimates at their reported analysis levels and may include dependent estimates from the same study. Findings without usable scalar estimates were reported narratively but were not included in the medians. Neither medians nor ranges are pooled effects or account for study size or precision. Negative session-duration values indicate shorter sessions under PS. RPE findings are summarized narratively in Section 3.4.1, Section 3.4.2 and Section 3.4.3. For crossover comparisons, 95% confidence intervals were not calculated when paired variability was unavailable. RPE = rating of perceived exertion; PS = paired set; TS = traditional set; ^a^ Volume load, completed repetitions, and training efficiency for the Paz companion study were reported by Paz et al. [17] (2019b; *n* = 22). ^b^ Session duration was reported by Paz et al. [42] (2019a; *n* = 13). The two reports were treated as companion reports from one underlying study but involved different sample sizes.

## Data Availability

No new primary data were generated in this systematic review. All data synthesized in the review were obtained from the cited publications and are reported in the manuscript and Supplementary Materials.

## Supplementary Materials

The following supporting information can be downloaded at: https://www.mdpi.com/article/10.3390/jfmk11030370/s1, Table S1: Database-Specific Systematic Search Strategies; Table S2: Reports Excluded After Full-Text Assessment and Reasons for Exclusion; Table S3: (A): Detailed Result-Level RoB 2 Assessments for Volume Load Outcomes; (B): Detailed Result-Level RoB 2 Assessments for Completed Repetition Outcomes; (C): Detailed Result-Level RoB 2 Assessments for Training Efficiency Outcomes.
