# Supplementary material for: Effects of Different Paired-Set Configurations and Rest Intervals on Acute Volume Load, Repetitions, and Training Efficiency: A Systematic Review
Source: J Funct Morphol Kinesiol. 2026 Sep 11;11(3):370. doi: 10.3390/jfmk11030370 (PMC13608641; doi:10.3390/jfmk11030370)
Supplement: Supplementary file 1 [file jfmk-11-00370-s001.zip › jfmk-4383138-supplementary.pdf]

Supplementary Material: Detailed Literature Search Strategies

Table S1. Database-Specific Systematic Search Strategies (Last Updated from 31 July to 5 August 2026).

Database	Search Date	Fields & Filters Applied	Search Syntax/Keywords	Number of research item
**PubMed/MEDLINE**	31 July 2026	MeSH terms and Title/Abstract [tiab] search.<br><br>Filters: English language.	(("Resistance Training"[MeSH Terms] OR "resistance train*"[Title/Abstract] OR "resistance exercise*"[Title/Abstract] OR "weight train*"[Title/Abstract] OR "weight lift*"[Title/Abstract] OR "strength train*"[Title/Abstract]) AND ("superset*"[Title/Abstract] OR "super set*"[Title/Abstract] OR "paired set*"[Title/Abstract] OR "compound set*"[Title/Abstract] OR "complex set*"[Title/Abstract] OR "reciprocal superset*"[Title/Abstract] OR "alternating set*"[Title/Abstract] OR "alternate set*"[Title/Abstract] OR "antagonist paired set*"[Title/Abstract] OR "agonist antagonist paired set*"[Title/Abstract] OR "agonist antagonist"[Title/Abstract:~0]) AND "English"[Language]) AND (english[Filter])	97
**SPORTDiscus (EBSCOhost)**	1 August 2026	Subject Terms (DE) and Title/Abstract (TI/AB) search.<br><br>Filters: English language.	(DE "RESISTANCE training" OR TI ("resistance train*" OR "resistance exercise*" OR "weight train*" OR "weight lift*" OR "strength train*") OR AB ("resistance train*" OR "resistance exercise*" OR "weight train*" OR "weight lift*" OR "strength train*"))AND(TI (superset* OR super-set* OR "super set*" OR "paired set*" OR paired-set* OR "compound set*" OR "complex set*" OR "reciprocal set*" OR "alternating set*" OR "alternate set*" OR (agonist N1 antagonist)) OR AB (superset* OR super-set* OR "super set*" OR "paired set*" OR paired-set* OR "compound set*" OR "complex set*" OR "reciprocal set*" OR "alternating set*" OR "alternate set*" OR (agonist N1 antagonist)))	144
**Scopus (Elsevier)**	31 July 2026	Title, Abstract, Keywords (TITLE-ABS-KEY) search.<br><br>Filters: Document Type: Article; Language: English.	TITLE-ABS-KEY (("resistance train*" OR "resistance exercise*" OR "weight train*" OR "strength train*") AND (superset* OR "super-set*" OR "paired set*" OR "paired-set*" OR "compound set*" OR "complex set*" OR "reciprocal superset*" OR "alternate peripheral superset*" OR "alternating set*" OR "antagonist paired set*" OR "agonist-antagonist paired set*" OR "agonist antagonist paired set*" OR "agonist-antagonist superset*" OR "agonist antagonist superset*" OR "agonist-antagonist resistance train*" OR "agonist antagonist resistance train*")) AND (LIMIT-TO (DOCTYPE , "ar")) AND (LIMIT-TO (LANGUAGE , "English"))	71

**Web of Science (Clarivate)**	5 August 2026	Filters: Article, Language: English.	TS=(("resistance train*" OR "resistance exercise*" OR "weight train*" OR "weight lift*" OR weightlift* OR "strength train*") AND (superset* OR "super set*" OR "super-set*" OR "paired set*" OR "paired-set*" OR "compound set*" OR "complex set*" OR "reciprocal superset*" OR "reciprocal set*" OR "alternate peripheral superset*" OR "alternating set*" OR "alternate set*" OR "antagonist paired set*" OR "agonist antagonist paired set*" OR "agonist-antagonist paired set*" OR "agonist antagonist superset*" OR "agonist-antagonist superset*" OR "agonist antagonist resistance train*" OR "agonist-antagonist resistance train*" OR (agonist NEAR/1 antagonist)))	209
**CINAHL (EBSCO)**	1 August 2026	Filters: Language: English.	(MH "Resistance Training+"OR TI ("resistance train*"OR "resistance exercise*" OR "weight train*" OR "weight lift*" OR "strength train*") OR AB ("resistance train*" OR "resistance exercise*" OR "weight train*" OR "weight lift*" OR "strength train*"))AND (TI (superset* OR "super set*" OR "super-set*" OR "paired set*" OR "paired-set*" OR "compound set*" OR "complex set*" OR "reciprocal superset*" OR "alternate peripheral superset*" OR "alternating set*" OR "alternate set*" OR "antagonist paired set*" OR "agonist antagonist paired set*" OR "agonist-antagonist paired set*" OR "agonist antagonist superset*" OR "agonist-antagonist superset*" OR "agonist antagonist resistance train*" OR "agonist-antagonist resistance train*" OR (agonist N0 antagonist)) OR AB (superset* OR "super set*" OR "super-set*" OR "paired set*" OR "paired-set*" OR "compound set*" OR "complex set*" OR "reciprocal superset*" OR "alternate peripheral superset*" OR "alternating set*" OR "alternate set*" OR "antagonist paired set*" OR "agonist antagonist paired set*" OR "agonist-antagonist paired set*" OR "agonist antagonist superset*" OR "agonist-antagonist superset*" OR "agonist antagonist resistance train*" OR "agonist-antagonist resistance train*" OR (agonist N0 antagonist)))	51
**Rehabilitation & Sport Medicine Source (EBSCO)**	1 August 2026	Filters: Language: English.	(MH "Resistance Training+"OR TI ("resistance train*"OR "resistance exercise*" OR "weight train*" OR "weight lift*" OR "strength train*") OR AB ("resistance train*" OR "resistance exercise*" OR "weight train*" OR "weight lift*" OR "strength train*"))AND (TI (superset* OR "super set*" OR "super-set*" OR "paired set*" OR "paired-set*" OR "compound set*" OR "complex set*" OR "reciprocal superset*" OR "alternate peripheral superset*" OR "alternating set*" OR "alternate set*" OR "antagonist paired set*" OR "agonist antagonist paired set*" OR "agonist-antagonist paired set*" OR "agonist antagonist superset*" OR "agonist-antagonist superset*" OR "agonist antagonist resistance train*" OR "agonist-antagonist resistance train*" OR (agonist N0 antagonist)) OR AB (superset* OR "super set*" OR "super-set*" OR "paired set*" OR "paired-set*" OR "compound set*" OR "complex set*" OR "reciprocal superset*" OR "alternate peripheral superset*" OR "alternating set*" OR "alternate set*" OR "antagonist paired set*" OR "agonist antagonist paired set*" OR "agonist-antagonist paired set*"	34

			OR "agonist antagonist superset*" OR "agonist-antagonist superset*" OR "agonist antagonist resistance train*" OR "agonist-antagonist resistance train*" OR (agonist N0 antagonist)))	
**Embase (Elsevier)**	31 July 2026	Emtree Terms (/exp) and Title/Abstract (:ti,ab) search.<br><br>Filters: Article, English language.	('resistance training'/exp OR 'resistance exercise'/exp OR 'weight lifting'/exp OR 'resistance train*':ti,ab,kw OR 'resistance exercise*':ti,ab,kw OR 'weight train*':ti,ab,kw OR 'weight lift*':ti,ab,kw OR weightlift*:ti,ab,kw OR 'strength train*':ti,ab,kw) AND (superset*:ti,ab,kw OR 'super set*':ti,ab,kw OR 'paired set*':ti,ab,kw OR 'compound set*':ti,ab,kw OR 'complex set*':ti,ab,kw OR 'reciprocal set*':ti,ab,kw OR 'alternating set*':ti,ab,kw OR 'alternate set*':ti,ab,kw OR ((agonist NEAR/1 antagonist):ti,ab,kw)) AND [english]/lim AND ([article]/lim OR [article in press]/lim)	96
**Cochrane CENTRAL**	31 July 2026	Content type: Trials (CENTRAL)<br><br>Search fields: Title, Abstract, Keywords	#1<br>MeSH descriptor: [Resistance Training] explode all trees<br>#2<br>((resistance NEXT train*) OR(resistance NEXT exercise*) OR (weight NEXT train*) OR (weight NEXT lift*) OR(strength NEXT train*)):ti,ab,kw<br>#3<br>#1 OR #2<br>#4<br>(superset* OR (super NEXT set*) OR(paired NEXT set*) OR (compound NEXT set*) OR(complex NEXT set*) OR (reciprocal NEXT superset*) OR (alternating NEXT set*) OR (alternate NEXT set*) OR (antagonist NEXT paired NEXT set*) OR (agonist NEXT antagonist NEXT paired NEXT set*) OR (agonist NEXT antagonist NEXT superset*)):ti,ab,kw<br>#5<br>#3 AND #4	35
			**TOTAL (with duplicate)**	737

Table S2. Reports Excluded After Full-Text Assessment and Reasons for Exclusion.

Excluded report	Identification route	Primary reason for exclusion	Specific rationale
**Belo et al. (2020) [38]**	Database searching	Wrong population	Participants were Brazilian jiu-jitsu athletes with prior resistance-training experience, but regular resistance-training practice during the preceding 6 months was an exclusion criterion. The sample therefore did not meet the review's resistance-trained population criterion.
**Arazi et al. (2015) [39]**	Database searching	Wrong comparator	Both experimental conditions used a super-set protocol and differed only in exercise order (bench press + lat pull-down vs. lat pull-down + bench press). No traditional-set comparator was included.
**Rovny (2006) [41]**	Database searching	Wrong study design	The publication is a book review of a general-audience resistance-training book, not a primary research report.
**Corrêa Neto et al. (2024) [40]**	Citation searching	Wrong intervention	The alternated condition used a four-exercise upper- and lower-body sequence with self-selected rest intervals between sets and exercises, rather than an eligible two-exercise paired-set structure.

Note. Each report is listed under the primary eligibility criterion that led to exclusion after full-text assessment.

Detailed Risk-of-Bias 2 Assessments

These supplementary tables present result-level Risk of Bias 2 (RoB 2) assessments and support for judgments, following Cochrane reporting guidance and the transparency recommendations described by Flemyng et al. [35]. Assessments were conducted for the specific results contributing to the three primary outcome categories assessed with RoB 2: volume load, completed repetitions, and training efficiency. Session duration was retained as a secondary/contextual outcome in the review and was not included in the outcome-specific RoB 2 assessment scope.

For crossover trials, the RoB 2 crossover-trial variant (18 March 2021) was used, including the domain addressing period and carryover effects; for parallel-group trials, the standard RoB 2 tool (22 August 2019) was used. The effect of interest was the effect of assignment to intervention. All judgments shown are final consensus judgments.

Table S3. (A) Detailed Result-Level RoB 2 Assessments for Volume Load Outcomes.

ID	Result identification	D1	S	D2	D3	D4	D5	Overall
**A003**	**Study/report:** Bentes et al., 2017 [16] **Comparison:** Superset vs traditional **Specific result:** Session total work / volume load **Timepoint:** End of acute session **Reported result:** Traditional 8277.38 ± 1399.90; superset 7594.90 ± 1624.51	**SC**	**SC**	**L**	**L**	**SC**	**SC**	**SC**

Support for judgment. D1: Randomization was reported, but sequence generation and/or allocation concealment were insufficiently described; baseline comparability by first-period sequence was not adequately reported. Evidence locator: PDF p.3.
S: The balance of intervention sequences and/or handling of period effects was insufficiently reported. The washout interval was probably adequate for an acute resistance-training response, but recovery before later periods was not directly verified.
D2: The intervention conditions were distinguishable to participants and personnel, but procedures were standardized and no trial-context deviations likely to affect the result were identified. The analysis retained the assigned condition or within-participant crossover comparison. Evidence locator: PDF pp.3-4.
D3: All or nearly all randomized participants appeared to contribute data for this specific result, with no meaningful missing outcome data identified.
D4: The result depended on counting technically valid repetitions and/or determining repetition failure by staff who could identify the intervention. Standardized technique and procedures reduced, but did not eliminate, potential measurement influence.
D5: No time-stamped trial registration, protocol, or statistical analysis plan was available to verify that the selected measure and analysis were prespecified before results were known. No direct selective reporting was identified, but selection among eligible measurements or analyses could not be excluded.
Overall: Overall risk of bias was judged as some concerns because at least one domain had some concerns and no domain was judged at high risk.

ID	Result identification	D1	S	D2	D3	D4	D5	Overall
**A007**	**Study/report:** Paz et al., 2014 [46] **Comparison:** Paired set vs traditional **Specific result:** Bench-press total volume load **Timepoint:** End of acute session **Reported result:** Paired set 1435.8 ± 218.4; traditional 1373.6 ± 198.1	**SC**	**SC**	**L**	**SC**	**SC**	**SC**	**SC**

Support for judgment. D1: Randomization was reported, but sequence generation and/or allocation concealment were insufficiently described; baseline comparability by first-period sequence was not adequately reported. Evidence locator: PDF p.4, PDF p.3.

S: The balance of intervention sequences and/or handling of period effects was insufficiently reported. The washout interval was probably adequate for an acute resistance-training response, but recovery before later periods was not directly verified. Evidence locator: PDF p.4, PDF p.5.

D2: The intervention conditions were distinguishable to participants and personnel, but procedures were standardized and no trial-context deviations likely to affect the result were identified. The analysis retained the assigned condition or within-participant crossover comparison. Evidence locator: PDF p.4, PDF pp.3-4, PDF p.5.

D3: Fourteen of 15 participants completed both protocols (93.3%). The reason for the missing participant was not reported and no sensitivity analysis was presented, so outcome-related missingness could not be excluded.

D4: The result depended on counting technically valid repetitions and/or determining repetition failure by staff who could identify the intervention. Standardized technique and procedures reduced, but did not eliminate, potential measurement influence. Evidence locator: PDF p.1, PDF pp.3-4, PDF p.4.

D5: No time-stamped trial registration, protocol, or statistical analysis plan was available to verify that the selected measure and analysis were prespecified before results were known. No direct selective reporting was identified, but selection among eligible measurements or analyses could not be excluded. Evidence locator: PDF p.1, PDF p.5.

Overall: Overall risk of bias was judged as some concerns because at least one domain had some concerns and no domain was judged at high risk.

ID	Result identification	D1	S	D2	D3	D4	D5	Overall
**A008**	**Study/report:** Paz et al., 2014 [46] **Comparison:** Paired set vs traditional **Specific result:** Seated-row total volume load **Timepoint:** End of acute session **Reported result:** Paired set 1625.1 ± 210.5; traditional 1493.7 ± 115.6	**SC**	**SC**	**L**	**SC**	**SC**	**SC**	**SC**

ID	Result identification	D1	S	D2	D3	D4	D5	Overall
**Support for judgment. D1:** Randomization was reported, but sequence generation and/or allocation concealment were insufficiently described; baseline comparability by first-period sequence was not adequately reported. Evidence locator: PDF p.4, PDF p.3.<br>**S:** The balance of intervention sequences and/or handling of period effects was insufficiently reported. The washout interval was probably adequate for an acute resistance-training response, but recovery before later periods was not directly verified. Evidence locator: PDF p.4, PDF p.5.<br>**D2:** The intervention conditions were distinguishable to participants and personnel, but procedures were standardized and no trial-context deviations likely to affect the result were identified. The analysis retained the assigned condition or within-participant crossover comparison. Evidence locator: PDF p.4, PDF pp.3-4, PDF p.5.<br>**D3:** Fourteen of 15 participants completed both protocols (93.3%). The reason for the missing participant was not reported and no sensitivity analysis was presented, so outcome-related missingness could not be excluded.<br>**D4:** The result depended on counting technically valid repetitions and/or determining repetition failure by staff who could identify the intervention. Standardized technique and procedures reduced, but did not eliminate, potential measurement influence. Evidence locator: PDF p.1, PDF pp.3-4, PDF p.4.<br>**D5:** No time-stamped trial registration, protocol, or statistical analysis plan was available to verify that the selected measure and analysis were prespecified before results were known. No direct selective reporting was identified, but selection among eligible measurements or analyses could not be excluded. Evidence locator: PDF p.1, PDF p.5.<br>**Overall:** Overall risk of bias was judged as some concerns because at least one domain had some concerns and no domain was judged at high risk.								
A009	**Study/report:** Paz et al., 2017 [47]<br>**Comparison:** Paired set vs traditional<br>**Specific result:** Bench-press total volume load<br>**Timepoint:** End of acute session<br>**Reported result:** PS 1328 ± 27.5 kg；TS 1188.4 ± 115 kg	SC	SC	L	L	SC	SC	SC
**Support for judgment. D1:** Randomization was reported, but sequence generation and/or allocation concealment were insufficiently described; baseline comparability by first-period sequence was not adequately reported.<br>**S:** The balance of intervention sequences and/or handling of period effects was insufficiently reported. The washout interval was probably adequate for an acute resistance-training response, but recovery before later periods was not directly verified.<br>**D2:** The intervention conditions were distinguishable to participants and personnel, but procedures were standardized and no trial-context deviations likely to affect the result were identified. The analysis retained the assigned condition or within-participant crossover comparison. Evidence locator: PDF p.3.<br>**D3:** All or nearly all randomized participants appeared to contribute data for this specific result, with no meaningful missing outcome data identified.<br>**D4:** The result depended on counting technically valid repetitions and/or determining repetition failure by staff who could identify the intervention. Standardized technique and procedures reduced, but did not eliminate, potential measurement influence. Evidence locator: PDF p.1, PDF p.5, PDF pp.2-3.<br>**D5:** No time-stamped trial registration, protocol, or statistical analysis plan was available to verify that the selected measure and analysis were prespecified before results were known. No direct selective reporting was identified, but selection among eligible measurements or analyses could not be excluded. Evidence locator: PDF p.5.<br>**Overall:** Overall risk of bias was judged as some concerns because at least one domain had some concerns and no domain was judged at high risk.								

ID	Result identification	D1	S	D2	D3	D4	D5	Overall
**A010**	**Study/report:** Paz et al., 2017 [47] **Comparison:** Paired set vs traditional **Specific result:** Seated-row total volume load **Timepoint:** End of acute session **Reported result:** PS 1249.4 ± 135.5 kg；TS 960.5 ± 100.1 kg	**SC**	**SC**	**L**	**L**	**SC**	**SC**	**SC**
**Support for judgment. D1:** Randomization was reported, but sequence generation and/or allocation concealment were insufficiently described; baseline comparability by first-period sequence was not adequately reported. **S:** The balance of intervention sequences and/or handling of period effects was insufficiently reported. The washout interval was probably adequate for an acute resistance-training response, but recovery before later periods was not directly verified. **D2:** The intervention conditions were distinguishable to participants and personnel, but procedures were standardized and no trial-context deviations likely to affect the result were identified. The analysis retained the assigned condition or within-participant crossover comparison. Evidence locator: PDF p.3. **D3:** All or nearly all randomized participants appeared to contribute data for this specific result, with no meaningful missing outcome data identified. **D4:** The result depended on counting technically valid repetitions and/or determining repetition failure by staff who could identify the intervention. Standardized technique and procedures reduced, but did not eliminate, potential measurement influence. Evidence locator: PDF p.1, PDF p.5, PDF pp.2-3. **D5:** No time-stamped trial registration, protocol, or statistical analysis plan was available to verify that the selected measure and analysis were prespecified before results were known. No direct selective reporting was identified, but selection among eligible measurements or analyses could not be excluded. Evidence locator: PDF p.5. **Overall:** Overall risk of bias was judged as some concerns because at least one domain had some concerns and no domain was judged at high risk.								
**A018**	**Study/report:** Paz et al., 2019b [17] **Comparison:** Paired set vs traditional **Specific result:** Session-total volume load **Timepoint:** End of acute session **Reported result:** Traditional 7356.8 ± 2279.7; paired set 7936.2 ± 2439.6	**SC**	**SC**	**L**	**L**	**SC**	**SC**	**SC**

ID	Result identification	D1	S	D2	D3	D4	D5	Overall
**Support for judgment. D1:** Randomization was reported, but sequence generation and/or allocation concealment were insufficiently described; baseline comparability by first-period sequence was not adequately reported. Evidence locator: PDF p.3.<br>**S:** The balance of intervention sequences and/or handling of period effects was insufficiently reported. The washout interval was probably adequate for an acute resistance-training response, but recovery before later periods was not directly verified. Evidence locator: PDF p.5.<br>**D2:** The intervention conditions were distinguishable to participants and personnel, but procedures were standardized and no trial-context deviations likely to affect the result were identified. The analysis retained the assigned condition or within-participant crossover comparison. Evidence locator: PDF p.3, PDF p.4, PDF p.5.<br>**D3:** All or nearly all randomized participants appeared to contribute data for this specific result, with no meaningful missing outcome data identified.<br>**D4:** The result depended on counting technically valid repetitions and/or determining repetition failure by staff who could identify the intervention. Standardized technique and procedures reduced, but did not eliminate, potential measurement influence. Evidence locator: PDF p.3, PDF pp.3-4, PDF p.4.<br>**D5:** No time-stamped trial registration, protocol, or statistical analysis plan was available to verify that the selected measure and analysis were prespecified before results were known. No direct selective reporting was identified, but selection among eligible measurements or analyses could not be excluded. Evidence locator: PDF p.3, PDF p.5, PDF p.6.<br>**Overall:** Overall risk of bias was judged as some concerns because at least one domain had some concerns and no domain was judged at high risk.								
**A021**	**Study/report:** Paz et al., 2019b [17]<br>**Comparison:** Superset vs traditional<br>**Specific result:** Session-total volume load<br>**Timepoint:** End of acute session<br>**Reported result:** Traditional 7356.8 ± 2279.7; superset 8063.2 ± 2270.5	**SC**	**SC**	**L**	**L**	**SC**	**SC**	**SC**
**Support for judgment. D1:** Randomization was reported, but sequence generation and/or allocation concealment were insufficiently described; baseline comparability by first-period sequence was not adequately reported. Evidence locator: PDF p.1, PDF p.2, PDF p.3.<br>**S:** The balance of intervention sequences and/or handling of period effects was insufficiently reported. The washout interval was probably adequate for an acute resistance-training response, but recovery before later periods was not directly verified. Evidence locator: PDF p.3, PDF p.5, PDF p.4.<br>**D2:** The intervention conditions were distinguishable to participants and personnel, but procedures were standardized and no trial-context deviations likely to affect the result were identified. The analysis retained the assigned condition or within-participant crossover comparison. Evidence locator: PDF p.3, PDF p.4, PDF p.5.<br>**D3:** All or nearly all randomized participants appeared to contribute data for this specific result, with no meaningful missing outcome data identified. Evidence locator: PDF p.1, PDF p.5, PDF p.6.<br>**D4:** The result depended on counting technically valid repetitions and/or determining repetition failure by staff who could identify the intervention. Standardized technique and procedures reduced, but did not eliminate, potential measurement influence. Evidence locator: PDF p.3, PDF pp.3-4, PDF p.4.<br>**D5:** No time-stamped trial registration, protocol, or statistical analysis plan was available to verify that the selected measure and analysis were prespecified before results were known. No direct selective reporting was identified, but selection among eligible measurements or analyses could not be excluded. Evidence locator: PDF p.3, PDF p.5, PDF p.6.<br>**Overall:** Overall risk of bias was judged as some concerns because at least one domain had some concerns and no domain was judged at high risk.								

ID	Result identification	D1	S	D2	D3	D4	D5	Overall
A024	**Study/report:** Robbins et al., 2010a [18] **Comparison:** Complex paired set vs traditional **Specific result:** Bench-pull total volume load **Timepoint:** End of acute session **Reported result:** Complex 1034.70 kg; traditional 1094.29 kg	SC	L	L	L	SC	SC	SC

Support for judgment. D1: Randomization was reported, but sequence generation and/or allocation concealment were insufficiently described; baseline comparability by first-period sequence was not adequately reported. Evidence locator: PDF p.2, PDF p.3.
S: Counterbalancing/randomized order and the washout interval supported a low risk of period or carryover bias; no material carryover problem was identified, although sequence counts or pre-period recovery checks were not always fully reported. Evidence locator: PDF p.2, PDF p.3.
D2: The intervention conditions were distinguishable to participants and personnel, but procedures were standardized and no trial-context deviations likely to affect the result were identified. The analysis retained the assigned condition or within-participant crossover comparison. Evidence locator: PDF p.5-6, PDF p.3, PDF p.6.
D3: All or nearly all randomized participants appeared to contribute data for this specific result, with no meaningful missing outcome data identified. Evidence locator: PDF p.2, PDF p.5.
D4: The result depended on counting technically valid repetitions and/or determining repetition failure by staff who could identify the intervention. Standardized technique and procedures reduced, but did not eliminate, potential measurement influence. Evidence locator: PDF p.3, PDF p.2-5, PDF p.5.
D5: No time-stamped trial registration, protocol, or statistical analysis plan was available to verify that the selected measure and analysis were prespecified before results were known. No direct selective reporting was identified, but selection among eligible measurements or analyses could not be excluded. Evidence locator: PDF p.3-6, PDF p.6, PDF p.2.
Overall: Overall risk of bias was judged as some concerns because at least one domain had some concerns and no domain was judged at high risk.

ID	Result identification	D1	S	D2	D3	D4	D5	Overall
**A027**	**Study/report:** Robbins et al., 2010b [19] **Comparison:** Agonist–antagonist paired set vs traditional **Specific result:** Bench-pull total volume load **Timepoint:** End of acute session **Reported result:** Paired set 895.4 ± 163.7; traditional 738.1 ± 102.6	**SC**	**L**	**L**	**L**	**SC**	**SC**	**SC**

Support for judgment. D1: Randomization was reported, but sequence generation and/or allocation concealment were insufficiently described; baseline comparability by first-period sequence was not adequately reported. Evidence locator: PDF p.3.

S: Counterbalancing/randomized order and the washout interval supported a low risk of period or carryover bias; no material carryover problem was identified, although sequence counts or pre-period recovery checks were not always fully reported. Evidence locator: PDF p.3, PDF p.4.

D2: The intervention conditions were distinguishable to participants and personnel, but procedures were standardized and no trial-context deviations likely to affect the result were identified. The analysis retained the assigned condition or within-participant crossover comparison. Evidence locator: PDF p.3, PDF pp.3-5, PDF p.6.

D3: All or nearly all randomized participants appeared to contribute data for this specific result, with no meaningful missing outcome data identified. Evidence locator: PDF p.4.

D4: The result depended on counting technically valid repetitions and/or determining repetition failure by staff who could identify the intervention. Standardized technique and procedures reduced, but did not eliminate, potential measurement influence. Evidence locator: PDF p.4, PDF pp.3-5, PDF pp.4-5.

D5: No time-stamped trial registration, protocol, or statistical analysis plan was available to verify that the selected measure and analysis were prespecified before results were known. No direct selective reporting was identified, but selection among eligible measurements or analyses could not be excluded. Evidence locator: PDF pp.1-9, PDF p.4, PDF p.6.

Overall: Overall risk of bias was judged as some concerns because at least one domain had some concerns and no domain was judged at high risk.

ID	Result identification	D1	S	D2	D3	D4	D5	Overall
A028	**Study/report:** Robbins et al., 2010b [19] **Comparison:** Agonist–antagonist paired set vs traditional **Specific result:** Bench-press total volume load **Timepoint:** End of acute session **Reported result:** Paired set 930.4 ± 238.8; traditional 731.3 ± 220.2	**SC**	**L**	**L**	**L**	**SC**	**SC**	**SC**

Support for judgment. D1: Randomization was reported, but sequence generation and/or allocation concealment were insufficiently described; baseline comparability by first-period sequence was not adequately reported. Evidence locator: PDF p.3.

S: Counterbalancing/randomized order and the washout interval supported a low risk of period or carryover bias; no material carryover problem was identified, although sequence counts or pre-period recovery checks were not always fully reported. Evidence locator: PDF p.3, PDF p.4.

D2: The intervention conditions were distinguishable to participants and personnel, but procedures were standardized and no trial-context deviations likely to affect the result were identified. The analysis retained the assigned condition or within-participant crossover comparison. Evidence locator: PDF p.3, PDF pp.3-5, PDF p.6.

D3: All or nearly all randomized participants appeared to contribute data for this specific result, with no meaningful missing outcome data identified. Evidence locator: PDF p.4.

D4: The result depended on counting technically valid repetitions and/or determining repetition failure by staff who could identify the intervention. Standardized technique and procedures reduced, but did not eliminate, potential measurement influence. Evidence locator: PDF p.4, PDF pp.3-5, PDF pp.4-5.

D5: No time-stamped trial registration, protocol, or statistical analysis plan was available to verify that the selected measure and analysis were prespecified before results were known. No direct selective reporting was identified, but selection among eligible measurements or analyses could not be excluded. Evidence locator: PDF pp.1-9, PDF p.4, PDF p.6.

Overall: Overall risk of bias was judged as some concerns because at least one domain had some concerns and no domain was judged at high risk.

ID	Result identification	D1	S	D2	D3	D4	D5	Overall
A032	**Study/report:** Robbins et al., 2010c [20] **Comparison:** Paired set vs traditional **Specific result:** Bench-pull total volume load **Timepoint:** End of acute session **Reported result:** Paired set 1060.56 ± 467.34; traditional 1105.49 ± 448.38	SC	L	L	L	SC	SC	SC

Support for judgment. D1: Randomization was reported, but sequence generation and/or allocation concealment were insufficiently described; baseline comparability by first-period sequence was not adequately reported. Evidence locator: PDF p.2, PDF p.3.
S: Counterbalancing/randomized order and the washout interval supported a low risk of period or carryover bias; no material carryover problem was identified, although sequence counts or pre-period recovery checks were not always fully reported. Evidence locator: PDF p.2, PDF p.4, PDF p.3.
D2: The intervention conditions were distinguishable to participants and personnel, but procedures were standardized and no trial-context deviations likely to affect the result were identified. The analysis retained the assigned condition or within-participant crossover comparison. Evidence locator: PDF pp.2-3, PDF pp.2-5, PDF p.6.
D3: All or nearly all randomized participants appeared to contribute data for this specific result, with no meaningful missing outcome data identified. Evidence locator: PDF p.3, PDF p.5.
D4: The result depended on counting technically valid repetitions and/or determining repetition failure by staff who could identify the intervention. Standardized technique and procedures reduced, but did not eliminate, potential measurement influence. Evidence locator: PDF p.3, PDF pp.2-5, PDF p.5.
D5: No time-stamped trial registration, protocol, or statistical analysis plan was available to verify that the selected measure and analysis were prespecified before results were known. No direct selective reporting was identified, but selection among eligible measurements or analyses could not be excluded. Evidence locator: PDF pp.1-9, PDF p.5, PDF p.6.
Overall: Overall risk of bias was judged as some concerns because at least one domain had some concerns and no domain was judged at high risk.

ID	Result identification	D1	S	D2	D3	D4	D5	Overall
A033	**Study/report:** Robbins et al., 2010c [20] **Comparison:** Paired set vs traditional **Specific result:** Bench-press total volume load **Timepoint:** End of acute session **Reported result:** Paired set 1039.58 ± 485.16; traditional 1107.47 ± 660.25	SC	L	L	L	SC	SC	SC

ID	Result identification	D1	S	D2	D3	D4	D5	Overall

Support for judgment. D1: Randomization was reported, but sequence generation and/or allocation concealment were insufficiently described; baseline comparability by first-period sequence was not adequately reported. Evidence locator: PDF p.2, PDF p.3.
S: Counterbalancing/randomized order and the washout interval supported a low risk of period or carryover bias; no material carryover problem was identified, although sequence counts or pre-period recovery checks were not always fully reported. Evidence locator: PDF p.2, PDF p.4, PDF p.3.
D2: The intervention conditions were distinguishable to participants and personnel, but procedures were standardized and no trial-context deviations likely to affect the result were identified. The analysis retained the assigned condition or within-participant crossover comparison. Evidence locator: PDF pp.2-3, PDF pp.2-5, PDF p.6.
D3: All or nearly all randomized participants appeared to contribute data for this specific result, with no meaningful missing outcome data identified. Evidence locator: PDF p.3, PDF p.5.
D4: The result depended on counting technically valid repetitions and/or determining repetition failure by staff who could identify the intervention. Standardized technique and procedures reduced, but did not eliminate, potential measurement influence. Evidence locator: PDF p.3, PDF pp.2-5, PDF p.5.
D5: No time-stamped trial registration, protocol, or statistical analysis plan was available to verify that the selected measure and analysis were prespecified before results were known. No direct selective reporting was identified, but selection among eligible measurements or analyses could not be excluded. Evidence locator: PDF pp.1-9, PDF p.5, PDF p.6.
Overall: Overall risk of bias was judged as some concerns because at least one domain had some concerns and no domain was judged at high risk.

ID	Result identification	D1	S	D2	D3	D4	D5	Overall
**A039**	**Study/report:** Nasser et al., 2019 [43] **Comparison:** APS vs traditional **Specific result:** Session-total volume load **Timepoint:** End of acute session **Reported result:** APS 6930.5 ± 458.1 kg; traditional 6092.5 ± 433.0 kg	**SC**	**L**	**L**	**L**	**SC**	**SC**	**SC**

Support for judgment. D1: Randomization was reported, but sequence generation and/or allocation concealment were insufficiently described; baseline comparability by first-period sequence was not adequately reported. Evidence locator: PDF pp.18-19, PDF p.18.
S: Counterbalancing/randomized order and the washout interval supported a low risk of period or carryover bias; no material carryover problem was identified, although sequence counts or pre-period recovery checks were not always fully reported. Evidence locator: PDF pp.17-19, PDF pp.18-19.
D2: The intervention conditions were distinguishable to participants and personnel, but procedures were standardized and no trial-context deviations likely to affect the result were identified. The analysis retained the assigned condition or within-participant crossover comparison. Evidence locator: PDF pp.17-19, PDF pp.18-19, PDF p.19.
D3: All or nearly all randomized participants appeared to contribute data for this specific result, with no meaningful missing outcome data identified. Evidence locator: PDF p.17, PDF p.20.
D4: The result depended on counting technically valid repetitions and/or determining repetition failure by staff who could identify the intervention. Standardized technique and procedures reduced, but did not eliminate, potential measurement influence. Evidence locator: PDF pp.19-20, PDF p.19.
D5: No time-stamped trial registration, protocol, or statistical analysis plan was available to verify that the selected measure and analysis were prespecified before results were known. No direct selective reporting was identified, but selection among eligible measurements or analyses could not be excluded. Evidence locator: PDF pp.17-22, PDF p.20, PDF p.19.
Overall: Overall risk of bias was judged as some concerns because at least one domain had some concerns and no domain was judged at high risk.

ID	Result identification	D1	S	D2	D3	D4	D5	Overall
**A045**	**Study/report:** Nasser et al., 2019 [43] **Comparison:** PAL vs traditional **Specific result:** Session-total volume load **Timepoint:** End of acute session **Reported result:** PAL 7239.2 ± 458.1 kg; traditional 6092.5 ± 433.0 kg	**SC**	**L**	**L**	**L**	**SC**	**SC**	**SC**

Support for judgment. D1: Randomization was reported, but sequence generation and/or allocation concealment were insufficiently described; baseline comparability by first-period sequence was not adequately reported. Evidence locator: PDF pp.18-19, PDF p.18.

S: Counterbalancing/randomized order and the washout interval supported a low risk of period or carryover bias; no material carryover problem was identified, although sequence counts or pre-period recovery checks were not always fully reported. Evidence locator: PDF pp.17-19, PDF pp.18-19.

D2: The intervention conditions were distinguishable to participants and personnel, but procedures were standardized and no trial-context deviations likely to affect the result were identified. The analysis retained the assigned condition or within-participant crossover comparison. Evidence locator: PDF pp.17-19, PDF pp.18-19, PDF p.19.

D3: All or nearly all randomized participants appeared to contribute data for this specific result, with no meaningful missing outcome data identified. Evidence locator: PDF p.17, PDF p.20.

D4: The result depended on counting technically valid repetitions and/or determining repetition failure by staff who could identify the intervention. Standardized technique and procedures reduced, but did not eliminate, potential measurement influence. Evidence locator: PDF pp.19-20, PDF p.19.

D5: No time-stamped trial registration, protocol, or statistical analysis plan was available to verify that the selected measure and analysis were prespecified before results were known. No direct selective reporting was identified, but selection among eligible measurements or analyses could not be excluded. Evidence locator: PDF pp.17-22, PDF p.20, PDF p.19.

Overall: Overall risk of bias was judged as some concerns because at least one domain had some concerns and no domain was judged at high risk.

ID	Result identification	D1	S	D2	D3	D4	D5	Overall
A061	**Study/report:** Yamazaki et al., 2025 [44] **Comparison:** Alternating vs 4-min traditional **Specific result:** Bench-press total volume load **Timepoint:** End of acute session **Reported result:** alternating 1911.3 ± 453.9; 4-minute traditional 1668.3 ± 365.9 kg	SC	SC	L	L	SC	SC	SC

Support for judgment. D1: Randomization was reported, but sequence generation and/or allocation concealment were insufficiently described; baseline comparability by first-period sequence was not adequately reported. Evidence locator: PDF p.2.

S: The balance of intervention sequences and/or handling of period effects was insufficiently reported. The washout interval was probably adequate for an acute resistance-training response, but recovery before later periods was not directly verified. Evidence locator: PDF p.2, PDF p.3.

D2: The intervention conditions were distinguishable to participants and personnel, but procedures were standardized and no trial-context deviations likely to affect the result were identified. The analysis retained the assigned condition or within-participant crossover comparison. Evidence locator: PDF pp.2-3, PDF pp.2-4.

D3: All or nearly all randomized participants appeared to contribute data for this specific result, with no meaningful missing outcome data identified. Evidence locator: PDF p.1.

D4: The result depended on counting technically valid repetitions and/or determining repetition failure by staff who could identify the intervention. Standardized technique and procedures reduced, but did not eliminate, potential measurement influence. Evidence locator: PDF pp.2-3, PDF p.3, PDF p.4.

D5: No time-stamped trial registration, protocol, or statistical analysis plan was available to verify that the selected measure and analysis were prespecified before results were known. No direct selective reporting was identified, but selection among eligible measurements or analyses could not be excluded. Evidence locator: PDF pp.1-6, PDF p.2, PDF p.1.

Overall: Overall risk of bias was judged as some concerns because at least one domain had some concerns and no domain was judged at high risk.

ID	Result identification	D1	S	D2	D3	D4	D5	Overall
**A062**	**Study/report:** Yamazaki et al., 2025 [44]<br>**Comparison:** Alternating vs 4-min traditional<br>**Specific result:** Back-squat total volume load<br>**Timepoint:** End of acute session<br>**Reported result:** alternating 2777.7 ± 814.0; 4-minute traditional 2336.7 ± 618.5 kg	**SC**	**SC**	**L**	**L**	**SC**	**SC**	**SC**

Support for judgment. D1: Randomization was reported, but sequence generation and/or allocation concealment were insufficiently described; baseline comparability by first-period sequence was not adequately reported. Evidence locator: PDF p.2.

S: The balance of intervention sequences and/or handling of period effects was insufficiently reported. The washout interval was probably adequate for an acute resistance-training response, but recovery before later periods was not directly verified. Evidence locator: PDF p.2, PDF p.3.

D2: The intervention conditions were distinguishable to participants and personnel, but procedures were standardized and no trial-context deviations likely to affect the result were identified. The analysis retained the assigned condition or within-participant crossover comparison. Evidence locator: PDF pp.2-3, PDF pp.2-4.

D3: All or nearly all randomized participants appeared to contribute data for this specific result, with no meaningful missing outcome data identified. Evidence locator: PDF p.1.

D4: The result depended on counting technically valid repetitions and/or determining repetition failure by staff who could identify the intervention. Standardized technique and procedures reduced, but did not eliminate, potential measurement influence. Evidence locator: PDF pp.2-3, PDF p.3, PDF p.4.

D5: No time-stamped trial registration, protocol, or statistical analysis plan was available to verify that the selected measure and analysis were prespecified before results were known. No direct selective reporting was identified, but selection among eligible measurements or analyses could not be excluded. Evidence locator: PDF pp.1-6, PDF p.2, PDF p.1.

Overall: Overall risk of bias was judged as some concerns because at least one domain had some concerns and no domain was judged at high risk.

ID	Result identification	D1	S	D2	D3	D4	D5	Overall
**A066**	**Study/report:** Yamazaki et al., 2025 [44] **Comparison:** Alternating vs 8-min traditional **Specific result:** Bench-press total volume load **Timepoint:** End of acute session **Reported result:** alternating 1911.3 ± 453.9; 8-minute traditional 2164.0 ± 519.1 kg	**SC**	**SC**	**L**	**L**	**SC**	**SC**	**SC**

Support for judgment. D1: Randomization was reported, but sequence generation and/or allocation concealment were insufficiently described; baseline comparability by first-period sequence was not adequately reported. Evidence locator: PDF p.2.

S: The balance of intervention sequences and/or handling of period effects was insufficiently reported. The washout interval was probably adequate for an acute resistance-training response, but recovery before later periods was not directly verified. Evidence locator: PDF p.2, PDF p.3.

D2: The intervention conditions were distinguishable to participants and personnel, but procedures were standardized and no trial-context deviations likely to affect the result were identified. The analysis retained the assigned condition or within-participant crossover comparison. Evidence locator: PDF pp.2-3, PDF pp.2-4.

D3: All or nearly all randomized participants appeared to contribute data for this specific result, with no meaningful missing outcome data identified. Evidence locator: PDF p.1.

D4: The result depended on counting technically valid repetitions and/or determining repetition failure by staff who could identify the intervention. Standardized technique and procedures reduced, but did not eliminate, potential measurement influence. Evidence locator: PDF pp.2-3, PDF p.3, PDF p.4.

D5: No time-stamped trial registration, protocol, or statistical analysis plan was available to verify that the selected measure and analysis were prespecified before results were known. No direct selective reporting was identified, but selection among eligible measurements or analyses could not be excluded. Evidence locator: PDF pp.1-6, PDF p.2, PDF p.1.

Overall: Overall risk of bias was judged as some concerns because at least one domain had some concerns and no domain was judged at high risk.

ID	Result identification	D1	S	D2	D3	D4	D5	Overall
**A067**	**Study/report:** Yamazaki et al., 2025 [44] **Comparison:** Alternating vs 8-min traditional **Specific result:** Back-squat total volume load **Timepoint:** End of acute session **Reported result:** alternating 2777.7 ± 814.0; 8-minute traditional 2899.2 ± 471.9 kg	**SC**	**SC**	**L**	**L**	**SC**	**SC**	**SC**

Support for judgment. D1: Randomization was reported, but sequence generation and/or allocation concealment were insufficiently described; baseline comparability by first-period sequence was not adequately reported. Evidence locator: PDF p.2.

S: The balance of intervention sequences and/or handling of period effects was insufficiently reported. The washout interval was probably adequate for an acute resistance-training response, but recovery before later periods was not directly verified. Evidence locator: PDF p.2, PDF p.3.

D2: The intervention conditions were distinguishable to participants and personnel, but procedures were standardized and no trial-context deviations likely to affect the result were identified. The analysis retained the assigned condition or within-participant crossover comparison. Evidence locator: PDF pp.2-3, PDF pp.2-4.

D3: All or nearly all randomized participants appeared to contribute data for this specific result, with no meaningful missing outcome data identified. Evidence locator: PDF p.1.

D4: The result depended on counting technically valid repetitions and/or determining repetition failure by staff who could identify the intervention. Standardized technique and procedures reduced, but did not eliminate, potential measurement influence. Evidence locator: PDF pp.2-3, PDF p.3, PDF p.4.

D5: No time-stamped trial registration, protocol, or statistical analysis plan was available to verify that the selected measure and analysis were prespecified before results were known. No direct selective reporting was identified, but selection among eligible measurements or analyses could not be excluded. Evidence locator: PDF pp.1-6, PDF p.2, PDF p.1.

Overall: Overall risk of bias was judged as some concerns because at least one domain had some concerns and no domain was judged at high risk.

Note. D1 = bias arising from the randomization process; S = bias arising from period and carryover effects; D2 = bias due to deviations from intended interventions; D3 = bias due to missing outcome data; D4 = bias in measurement of the outcome; D5 = bias in selection of the reported result. L = low risk of bias; SC = some concerns; H = high risk of bias; N/A = not applicable. Support for judgment is a concise English synthesis of the final consensus rationale. Evidence locators are retained where available. Complete consensus responses to the RoB 2 signalling questions and the full source-located rationale should be supplied with the final consensus workbook as supplementary data.

Table S3. (B) Detailed Result-Level RoB 2 Assessments for Completed Repetition Outcomes.

<table>
<tr><th>ID</th><th>Result identification</th><th>D1</th><th>S</th><th>D2</th><th>D3</th><th>D4</th><th>D5</th><th>Overall</th></tr>
<tr><td>A001</td><td>Study/report: Andersen et al., 2022 [14]
Comparison: Superset vs traditional
Specific result: Session-total completed repetitions
Timepoint: End of acute session
Reported result: Traditional 227 ± 29; superset 218 ± 26</td><td>SC</td><td>L</td><td>L</td><td>L</td><td>SC</td><td>SC</td><td>SC</td></tr>
<tr><td colspan="9">Support for judgment. D1: Randomization was reported, but sequence generation and/or allocation concealment were insufficiently described; baseline comparability by first-period sequence was not adequately reported. Evidence locator: PDF p.2.
S: Counterbalancing/randomized order and the washout interval supported a low risk of period or carryover bias; no material carryover problem was identified, although sequence counts or pre-period recovery checks were not always fully reported.
D2: The intervention conditions were distinguishable to participants and personnel, but procedures were standardized and no trial-context deviations likely to affect the result were identified. The analysis retained the assigned condition or within-participant crossover comparison.
D3: All or nearly all randomized participants appeared to contribute data for this specific result, with no meaningful missing outcome data identified.
D4: The result depended on counting technically valid repetitions and/or determining repetition failure by staff who could identify the intervention. Standardized technique and procedures reduced, but did not eliminate, potential measurement influence.
D5: No time-stamped trial registration, protocol, or statistical analysis plan was available to verify that the selected measure and analysis were prespecified before results were known. No direct selective reporting was identified, but selection among eligible measurements or analyses could not be excluded.
Overall: Overall risk of bias was judged as some concerns because at least one domain had some concerns and no domain was judged at high risk.</td></tr>
<tr><td>A019</td><td>Study/report: Paz et al., 2019b [17]
Comparison: Paired set vs traditional
Specific result: Session-total completed repetitions
Timepoint: End of acute session
Reported result: Traditional 133.6 ± 23.0; paired set 142.0 ± 23.4</td><td>SC</td><td>SC</td><td>L</td><td>L</td><td>SC</td><td>SC</td><td>SC</td></tr>
</table>

ID	Result identification	D1	S	D2	D3	D4	D5	Overall
**Support for judgment. D1:** Randomization was reported, but sequence generation and/or allocation concealment were insufficiently described; baseline comparability by first-period sequence was not adequately reported. Evidence locator: PDF p.1, PDF p.2, PDF p.3.<br>**S:** The balance of intervention sequences and/or handling of period effects was insufficiently reported. The washout interval was probably adequate for an acute resistance-training response, but recovery before later periods was not directly verified. Evidence locator: PDF p.3, PDF p.5, PDF p.4.<br>**D2:** The intervention conditions were distinguishable to participants and personnel, but procedures were standardized and no trial-context deviations likely to affect the result were identified. The analysis retained the assigned condition or within-participant crossover comparison. Evidence locator: PDF p.3, PDF p.4, PDF p.5.<br>**D3:** All or nearly all randomized participants appeared to contribute data for this specific result, with no meaningful missing outcome data identified. Evidence locator: PDF p.1, PDF p.5, PDF p.6.<br>**D4:** The result depended on counting technically valid repetitions and/or determining repetition failure by staff who could identify the intervention. Standardized technique and procedures reduced, but did not eliminate, potential measurement influence. Evidence locator: PDF p.3, PDF p.4.<br>**D5:** No time-stamped trial registration, protocol, or statistical analysis plan was available to verify that the selected measure and analysis were prespecified before results were known. No direct selective reporting was identified, but selection among eligible measurements or analyses could not be excluded. Evidence locator: PDF p.3, PDF p.5, PDF p.6.<br>**Overall:** Overall risk of bias was judged as some concerns because at least one domain had some concerns and no domain was judged at high risk.								
**A022**	**Study/report:** Paz et al., 2019b [17]<br>**Comparison:** Superset vs traditional<br>**Specific result:** Session-total completed repetitions<br>**Timepoint:** End of acute session<br>**Reported result:** Traditional 133.6 ± 23.0; superset 144.6 ± 16.3	**SC**	**SC**	**L**	**L**	**SC**	**SC**	**SC**
**Support for judgment. D1:** Randomization was reported, but sequence generation and/or allocation concealment were insufficiently described; baseline comparability by first-period sequence was not adequately reported. Evidence locator: PDF p.1, PDF p.2, PDF p.3.<br>**S:** The balance of intervention sequences and/or handling of period effects was insufficiently reported. The washout interval was probably adequate for an acute resistance-training response, but recovery before later periods was not directly verified. Evidence locator: PDF p.3, PDF p.5, PDF p.4.<br>**D2:** The intervention conditions were distinguishable to participants and personnel, but procedures were standardized and no trial-context deviations likely to affect the result were identified. The analysis retained the assigned condition or within-participant crossover comparison. Evidence locator: PDF p.3, PDF p.4, PDF p.5.<br>**D3:** All or nearly all randomized participants appeared to contribute data for this specific result, with no meaningful missing outcome data identified. Evidence locator: PDF p.1, PDF p.5, PDF p.6.<br>**D4:** The result depended on counting technically valid repetitions and/or determining repetition failure by staff who could identify the intervention. Standardized technique and procedures reduced, but did not eliminate, potential measurement influence. Evidence locator: PDF p.3, PDF p.4.<br>**D5:** No time-stamped trial registration, protocol, or statistical analysis plan was available to verify that the selected measure and analysis were prespecified before results were known. No direct selective reporting was identified, but selection among eligible measurements or analyses could not be excluded. Evidence locator: PDF pp.1-12, PDF p.5, PDF p.3.<br>**Overall:** Overall risk of bias was judged as some concerns because at least one domain had some concerns and no domain was judged at high risk.								
**A041**	**Study/report:** Nasser et al., 2019 [43]	**SC**	**L**	**L**	**L**	**SC**	**SC**	**SC**

ID	Result identification	D1	S	D2	D3	D4	D5	Overall
	**Comparison:** APS vs traditional<br>**Specific result:** Biceps curl total completed repetitions<br>**Timepoint:** End of acute session<br>**Reported result:** APS 38.7 ± 5.8; traditional 34.7 ± 7.6 repetitions							
**Support for judgment. D1:** Randomization was reported, but sequence generation and/or allocation concealment were insufficiently described; baseline comparability by first-period sequence was not adequately reported. Evidence locator: PDF pp.18-19, PDF p.18.<br>**S:** Counterbalancing/randomized order and the washout interval supported a low risk of period or carryover bias; no material carryover problem was identified, although sequence counts or pre-period recovery checks were not always fully reported. Evidence locator: PDF pp.17-19, PDF pp.18-19.<br>**D2:** The intervention conditions were distinguishable to participants and personnel, but procedures were standardized and no trial-context deviations likely to affect the result were identified. The analysis retained the assigned condition or within-participant crossover comparison. Evidence locator: PDF pp.17-19, PDF pp.18-19, PDF p.19.<br>**D3:** All or nearly all randomized participants appeared to contribute data for this specific result, with no meaningful missing outcome data identified. Evidence locator: PDF p.17, PDF p.20.<br>**D4:** The result depended on counting technically valid repetitions and/or determining repetition failure by staff who could identify the intervention. Standardized technique and procedures reduced, but did not eliminate, potential measurement influence. Evidence locator: PDF pp.19-20, PDF p.19.<br>**D5:** No time-stamped trial registration, protocol, or statistical analysis plan was available to verify that the selected measure and analysis were prespecified before results were known. No direct selective reporting was identified, but selection among eligible measurements or analyses could not be excluded. Evidence locator: PDF pp.17-22, PDF p.20, PDF p.19.<br>**Overall:** Overall risk of bias was judged as some concerns because at least one domain had some concerns and no domain was judged at high risk.								
**A042**	**Study/report:** Nasser et al., 2019 [43]<br>**Comparison:** APS vs traditional<br>**Specific result:** Triceps pushdown total completed repetitions<br>**Timepoint:** End of acute session<br>**Reported result:** APS 40.5 ± 5.2; traditional 34.2 ± 5.5 repetitions	**SC**	**L**	**L**	**L**	**SC**	**SC**	**SC**
**Support for judgment. D1:** Randomization was reported, but sequence generation and/or allocation concealment were insufficiently described; baseline comparability by first-period sequence was not adequately reported. Evidence locator: PDF pp.18-19, PDF p.18.								

<table>
<tr><th>ID</th><th>Result identifica-tion</th><th>D1</th><th>S</th><th>D2</th><th>D3</th><th>D4</th><th>D5</th><th>Overall</th></tr>
<tr><td colspan="9">S: Counterbalancing/randomized order and the washout interval supported a low risk of period or carryover bias; no material carryover problem was identified, although sequence counts or pre-period recovery checks were not always fully reported. Evidence locator: PDF pp.17-19, PDF pp.18-19.
D2: The intervention conditions were distinguishable to participants and personnel, but procedures were standardized and no trial-context deviations likely to affect the result were identified. The analysis retained the assigned condition or within-participant crossover comparison. Evidence locator: PDF pp.17-19, PDF pp.18-19, PDF p.19.
D3: All or nearly all randomized participants appeared to contribute data for this specific result, with no meaningful missing outcome data identified. Evidence locator: PDF p.17, PDF p.20.
D4: The result depended on counting technically valid repetitions and/or determining repetition failure by staff who could identify the intervention. Standardized technique and procedures reduced, but did not eliminate, potential measurement influence. Evidence locator: PDF pp.19-20, PDF p.19.
D5: No time-stamped trial registration, protocol, or statistical analysis plan was available to verify that the selected measure and analysis were prespecified before results were known. No direct selective reporting was identified, but selection among eligible measurements or analyses could not be excluded. Evidence locator: PDF pp.17-22, PDF p.20, PDF p.19.
Overall: Overall risk of bias was judged as some concerns because at least one domain had some concerns and no domain was judged at high risk.</td></tr>
<tr><td>A043</td><td>Study/report: Nasser et al., 2019 [43]
Comparison: APS vs traditional
Specific result: Leg curl total completed repetitions
Timepoint: End of acute session
Reported result: APS 39.2 ± 7.4; traditional 35.2 ± 7.3 repetitions</td><td>SC</td><td>L</td><td>L</td><td>L</td><td>SC</td><td>SC</td><td>SC</td></tr>
<tr><td colspan="9">Support for judgment. D1: Randomization was reported, but sequence generation and/or allocation concealment were insufficiently described; baseline comparability by first-period sequence was not adequately reported. Evidence locator: PDF pp.18-19, PDF p.18.
S: Counterbalancing/randomized order and the washout interval supported a low risk of period or carryover bias; no material carryover problem was identified, although sequence counts or pre-period recovery checks were not always fully reported. Evidence locator: PDF pp.17-19, PDF pp.18-19.
D2: The intervention conditions were distinguishable to participants and personnel, but procedures were standardized and no trial-context deviations likely to affect the result were identified. The analysis retained the assigned condition or within-participant crossover comparison. Evidence locator: PDF pp.17-19, PDF pp.18-19, PDF p.19.
D3: All or nearly all randomized participants appeared to contribute data for this specific result, with no meaningful missing outcome data identified. Evidence locator: PDF p.17, PDF p.20.
D4: The result depended on counting technically valid repetitions and/or determining repetition failure by staff who could identify the intervention. Standardized technique and procedures reduced, but did not eliminate, potential measurement influence. Evidence locator: PDF pp.19-20, PDF p.19.
D5: No time-stamped trial registration, protocol, or statistical analysis plan was available to verify that the selected measure and analysis were prespecified before results were known. No direct selective reporting was identified, but selection among eligible measurements or analyses could not be excluded. Evidence locator: PDF pp.17-22, PDF p.20, PDF p.19.
Overall: Overall risk of bias was judged as some concerns because at least one domain had some concerns and no domain was judged at high risk.</td></tr>
<tr><td>A044</td><td>Study/report: Nasser et al., 2019 [43]
Comparison: APS vs traditional</td><td>SC</td><td>L</td><td>L</td><td>L</td><td>SC</td><td>SC</td><td>SC</td></tr>
</table>

ID	Result identification	D1	S	D2	D3	D4	D5	Overall
	**Specific result:** Leg extension total completed repetitions<br>**Timepoint:** End of acute session<br>**Reported result:** APS 38.7 ± 4.4; traditional 33.8 ± 5.3 repetitions							

Support for judgment. D1: Randomization was reported, but sequence generation and/or allocation concealment were insufficiently described; baseline comparability by first-period sequence was not adequately reported. Evidence locator: PDF pp.18-19, PDF p.18.
S: Counterbalancing/randomized order and the washout interval supported a low risk of period or carryover bias; no material carryover problem was identified, although sequence counts or pre-period recovery checks were not always fully reported. Evidence locator: PDF pp.17-19, PDF pp.18-19.
D2: The intervention conditions were distinguishable to participants and personnel, but procedures were standardized and no trial-context deviations likely to affect the result were identified. The analysis retained the assigned condition or within-participant crossover comparison. Evidence locator: PDF pp.17-19, PDF pp.18-19, PDF p.19.
D3: All or nearly all randomized participants appeared to contribute data for this specific result, with no meaningful missing outcome data identified. Evidence locator: PDF p.17, PDF p.20.
D4: The result depended on counting technically valid repetitions and/or determining repetition failure by staff who could identify the intervention. Standardized technique and procedures reduced, but did not eliminate, potential measurement influence. Evidence locator: PDF pp.19-20, PDF p.19.
D5: No time-stamped trial registration, protocol, or statistical analysis plan was available to verify that the selected measure and analysis were prespecified before results were known. No direct selective reporting was identified, but selection among eligible measurements or analyses could not be excluded. Evidence locator: PDF pp.17-22, PDF p.20, PDF p.19.
Overall: Overall risk of bias was judged as some concerns because at least one domain had some concerns and no domain was judged at high risk.

ID	Result identification	D1	S	D2	D3	D4	D5	Overall
A047	**Study/report:** Nasser et al., 2019 [43]<br>**Comparison:** PAL vs traditional<br>**Specific result:** Biceps curl total completed repetitions<br>**Timepoint:** End of acute session<br>**Reported result:** PAL 40.1 ± 5.8; traditional 34.7 ± 7.6 repetitions	SC	L	L	L	SC	SC	SC

Support for judgment. D1: Randomization was reported, but sequence generation and/or allocation concealment were insufficiently described; baseline comparability by first-period sequence was not adequately reported. Evidence locator: PDF pp.18-19, PDF p.18.
S: Counterbalancing/randomized order and the washout interval supported a low risk of period or carryover bias; no material carryover problem was identified, although sequence counts or pre-period recovery checks were not always fully reported. Evidence locator: PDF pp.17-19, PDF pp.18-19.
D2: The intervention conditions were distinguishable to participants and personnel, but procedures were standardized and no trial-context deviations likely to affect the result were identified. The analysis retained the assigned condition or within-participant crossover comparison. Evidence locator: PDF pp.17-19, PDF pp.18-19, PDF p.19.

ID	Result identification	D1	S	D2	D3	D4	D5	Overall
**D3:** All or nearly all randomized participants appeared to contribute data for this specific result, with no meaningful missing outcome data identified. Evidence locator: PDF p.17, PDF p.20.<br>**D4:** The result depended on counting technically valid repetitions and/or determining repetition failure by staff who could identify the intervention. Standardized technique and procedures reduced, but did not eliminate, potential measurement influence. Evidence locator: PDF pp.19-20, PDF p.19.<br>**D5:** No time-stamped trial registration, protocol, or statistical analysis plan was available to verify that the selected measure and analysis were prespecified before results were known. No direct selective reporting was identified, but selection among eligible measurements or analyses could not be excluded. Evidence locator: PDF pp.17-22, PDF p.20, PDF p.19.<br>**Overall:** Overall risk of bias was judged as some concerns because at least one domain had some concerns and no domain was judged at high risk.								
**A048**	**Study/report:** Nasser et al., 2019 [43]<br>**Comparison:** PAL vs traditional<br>**Specific result:** Triceps pushdown total completed repetitions<br>**Timepoint:** End of acute session<br>**Reported result:** PAL 38.3 ± 6.7; traditional 34.2 ± 5.5 repetitions	**SC**	**L**	**L**	**L**	**SC**	**SC**	**SC**
**Support for judgment. D1:** Randomization was reported, but sequence generation and/or allocation concealment were insufficiently described; baseline comparability by first-period sequence was not adequately reported. Evidence locator: PDF pp.18-19, PDF p.18.<br>**S:** Counterbalancing/randomized order and the washout interval supported a low risk of period or carryover bias; no material carryover problem was identified, although sequence counts or pre-period recovery checks were not always fully reported. Evidence locator: PDF pp.17-19, PDF pp.18-19.<br>**D2:** The intervention conditions were distinguishable to participants and personnel, but procedures were standardized and no trial-context deviations likely to affect the result were identified. The analysis retained the assigned condition or within-participant crossover comparison. Evidence locator: PDF pp.17-19, PDF pp.18-19, PDF p.19.<br>**D3:** All or nearly all randomized participants appeared to contribute data for this specific result, with no meaningful missing outcome data identified. Evidence locator: PDF p.17, PDF p.20.<br>**D4:** The result depended on counting technically valid repetitions and/or determining repetition failure by staff who could identify the intervention. Standardized technique and procedures reduced, but did not eliminate, potential measurement influence. Evidence locator: PDF pp.19-20, PDF p.19.<br>**D5:** No time-stamped trial registration, protocol, or statistical analysis plan was available to verify that the selected measure and analysis were prespecified before results were known. No direct selective reporting was identified, but selection among eligible measurements or analyses could not be excluded. Evidence locator: PDF pp.17-22, PDF p.20, PDF p.19.<br>**Overall:** Overall risk of bias was judged as some concerns because at least one domain had some concerns and no domain was judged at high risk.								
**A049**	**Study/report:** Nasser et al., 2019 [43]<br>**Comparison:** PAL vs traditional<br>**Specific result:** Leg curl total completed repetitions	**SC**	**L**	**L**	**L**	**SC**	**SC**	**SC**

ID	Result identification	D1	S	D2	D3	D4	D5	Overall
	**Timepoint:** End of acute session<br>**Reported result:** PAL 44.6 ± 8.4; traditional 35.2 ± 7.3 repetitions							
**Support for judgment. D1:** Randomization was reported, but sequence generation and/or allocation concealment were insufficiently described; baseline comparability by first-period sequence was not adequately reported. Evidence locator: PDF pp.18-19, PDF p.18.<br>**S:** Counterbalancing/randomized order and the washout interval supported a low risk of period or carryover bias; no material carryover problem was identified, although sequence counts or pre-period recovery checks were not always fully reported. Evidence locator: PDF pp.17-19, PDF pp.18-19.<br>**D2:** The intervention conditions were distinguishable to participants and personnel, but procedures were standardized and no trial-context deviations likely to affect the result were identified. The analysis retained the assigned condition or within-participant crossover comparison. Evidence locator: PDF pp.17-19, PDF pp.18-19, PDF p.19.<br>**D3:** All or nearly all randomized participants appeared to contribute data for this specific result, with no meaningful missing outcome data identified. Evidence locator: PDF p.17, PDF p.20.<br>**D4:** The result depended on counting technically valid repetitions and/or determining repetition failure by staff who could identify the intervention. Standardized technique and procedures reduced, but did not eliminate, potential measurement influence. Evidence locator: PDF pp.19-20, PDF p.19.<br>**D5:** No time-stamped trial registration, protocol, or statistical analysis plan was available to verify that the selected measure and analysis were prespecified before results were known. No direct selective reporting was identified, but selection among eligible measurements or analyses could not be excluded. Evidence locator: PDF pp.17-22, PDF p.20, PDF p.19.<br>**Overall:** Overall risk of bias was judged as some concerns because at least one domain had some concerns and no domain was judged at high risk.								
**A050**	**Study/report:** Nasser et al., 2019 [43]<br>**Comparison:** PAL vs traditional<br>**Specific result:** Leg extension total completed repetitions<br>**Timepoint:** End of acute session<br>**Reported result:** PAL 39.3 ± 5.9; traditional 33.8 ± 5.3 repetitions	**SC**	**L**	**L**	**L**	**SC**	**SC**	**SC**
**Support for judgment. D1:** Randomization was reported, but sequence generation and/or allocation concealment were insufficiently described; baseline comparability by first-period sequence was not adequately reported. Evidence locator: PDF pp.18-19, PDF p.18.<br>**S:** Counterbalancing/randomized order and the washout interval supported a low risk of period or carryover bias; no material carryover problem was identified, although sequence counts or pre-period recovery checks were not always fully reported. Evidence locator: PDF pp.17-19, PDF pp.18-19.<br>**D2:** The intervention conditions were distinguishable to participants and personnel, but procedures were standardized and no trial-context deviations likely to affect the result were identified. The analysis retained the assigned condition or within-participant crossover comparison. Evidence locator: PDF pp.17-19, PDF pp.18-19, PDF p.19.<br>**D3:** All or nearly all randomized participants appeared to contribute data for this specific result, with no meaningful missing outcome data identified. Evidence locator: PDF p.17, PDF p.20.<br>**D4:** The result depended on counting technically valid repetitions and/or determining repetition failure by staff who could identify the intervention. Standardized technique and procedures reduced, but did not eliminate, potential measurement influence. Evidence locator: PDF pp.19-20, PDF p.19.								

ID	Result identification	D1	S	D2	D3	D4	D5	Overall
**D5:** No time-stamped trial registration, protocol, or statistical analysis plan was available to verify that the selected measure and analysis were prespecified before results were known. No direct selective reporting was identified, but selection among eligible measurements or analyses could not be excluded. Evidence locator: PDF pp.17-22, PDF p.20, PDF p.19. **Overall:** Overall risk of bias was judged as some concerns because at least one domain had some concerns and no domain was judged at high risk.								
**A051**	**Study/report:** Deminice et al., 2011 [45] **Comparison:** Alternating-pair circuit vs traditional **Specific result:** Average total repetitions per exercise **Timepoint:** End of acute session **Reported result:** alternating-pair circuit 29.3 ± 0.8; traditional 29.5 ± 0.7 repetitions	**SC**	**L**	**L**	**L**	**SC**	**SC**	**SC**
**Support for judgment. D1:** Randomization was reported, but sequence generation and/or allocation concealment were insufficiently described; baseline comparability by first-period sequence was not adequately reported. Evidence locator: PDF p.2, PDF p.3. **S:** Counterbalancing/randomized order and the washout interval supported a low risk of period or carryover bias; no material carryover problem was identified, although sequence counts or pre-period recovery checks were not always fully reported. Evidence locator: PDF p.2, PDF p.3, PDF pp.2-3. **D2:** The intervention conditions were distinguishable to participants and personnel, but procedures were standardized and no trial-context deviations likely to affect the result were identified. The analysis retained the assigned condition or within-participant crossover comparison. Evidence locator: PDF pp.2-3, PDF p.4. **D3:** All or nearly all randomized participants appeared to contribute data for this specific result, with no meaningful missing outcome data identified. Evidence locator: PDF pp.1-2, PDF p.4. **D4:** The result depended on counting technically valid repetitions and/or determining repetition failure by staff who could identify the intervention. Standardized technique and procedures reduced, but did not eliminate, potential measurement influence. Evidence locator: PDF pp.2-4, PDF p.4, PDF pp.2-3. **D5:** No time-stamped trial registration, protocol, or statistical analysis plan was available to verify that the selected measure and analysis were prespecified before results were known. No direct selective reporting was identified, but selection among eligible measurements or analyses could not be excluded. Evidence locator: PDF pp.1-7, PDF p.4, PDF p.1. **Overall:** Overall risk of bias was judged as some concerns because at least one domain had some concerns and no domain was judged at high risk.								
**A053**	**Study/report:** de Souza et al., 2017 [9] **Comparison:** Paired set vs traditional **Specific result:** Seated-row repetitions in sets 1–3 **Timepoint:** End of acute session	**SC**	**SC**	**L**	**L**	**SC**	**SC**	**SC**

ID	Result identification	D1	S	D2	D3	D4	D5	Overall
	**Reported result:** paired set [8.0 ± 0.0; 7.6 ± 0.5; 6.9 ± 0.3]; traditional [8.0 ± 0.0; 6.8 ± 0.4; 5.9 ± 0.7] repetitions per set							

Support for judgment. D1: Randomization was reported, but sequence generation and/or allocation concealment were insufficiently described; baseline comparability by first-period sequence was not adequately reported. Evidence locator: PDF p.3, PDF p.4.
S: The balance of intervention sequences and/or handling of period effects was insufficiently reported. The washout interval was probably adequate for an acute resistance-training response, but recovery before later periods was not directly verified. Evidence locator: PDF p.3, PDF p.4.
D2: The intervention conditions were distinguishable to participants and personnel, but procedures were standardized and no trial-context deviations likely to affect the result were identified. The analysis retained the assigned condition or within-participant crossover comparison. Evidence locator: PDF pp.3-4, PDF p.4.
D3: All or nearly all randomized participants appeared to contribute data for this specific result, with no meaningful missing outcome data identified. Evidence locator: PDF p.2, PDF p.4.
D4: The result depended on counting technically valid repetitions and/or determining repetition failure by staff who could identify the intervention. Standardized technique and procedures reduced, but did not eliminate, potential measurement influence. Evidence locator: PDF pp.3-4, PDF p.4, PDF p.3.
D5: No time-stamped trial registration, protocol, or statistical analysis plan was available to verify that the selected measure and analysis were prespecified before results were known. No direct selective reporting was identified, but selection among eligible measurements or analyses could not be excluded. Evidence locator: PDF pp.2-7, PDF p.4, PDF p.2.
Overall: Overall risk of bias was judged as some concerns because at least one domain had some concerns and no domain was judged at high risk.

ID	Result identification	D1	S	D2	D3	D4	D5	Overall
**A054**	**Study/report:** de Souza et al., 2017 [9] **Comparison:** Paired set vs traditional **Specific result:** Bench-press repetitions in sets 1–3 **Timepoint:** End of acute session **Reported result:** paired set [8.0 ± 0.0; 7.8 ± 0.4; 7.0 ± 0.0]; traditional [8.0 ± 0.0; 6.8 ± 0.4; 5.9 ± 0.7] repetitions per set	**SC**	**SC**	**L**	**L**	**SC**	**SC**	**SC**

Support for judgment. D1: Randomization was reported, but sequence generation and/or allocation concealment were insufficiently described; baseline comparability by first-period sequence was not adequately reported. Evidence locator: PDF p.3, PDF p.4.
S: The balance of intervention sequences and/or handling of period effects was insufficiently reported. The washout interval was probably adequate for an acute resistance-training response, but recovery before later periods was not directly verified. Evidence locator: PDF p.3, PDF p.4.

ID	Result identification	D1	S	D2	D3	D4	D5	Overall
**D2:** The intervention conditions were distinguishable to participants and personnel, but procedures were standardized and no trial-context deviations likely to affect the result were identified. The analysis retained the assigned condition or within-participant crossover comparison. Evidence locator: PDF pp.3-4, PDF p.4. **D3:** All or nearly all randomized participants appeared to contribute data for this specific result, with no meaningful missing outcome data identified. Evidence locator: PDF p.2, PDF p.4. **D4:** The result depended on counting technically valid repetitions and/or determining repetition failure by staff who could identify the intervention. Standardized technique and procedures reduced, but did not eliminate, potential measurement influence. Evidence locator: PDF pp.3-4, PDF p.4, PDF p.3. **D5:** No time-stamped trial registration, protocol, or statistical analysis plan was available to verify that the selected measure and analysis were prespecified before results were known. No direct selective reporting was identified, but selection among eligible measurements or analyses could not be excluded. Evidence locator: PDF pp.2-7, PDF p.4, PDF p.2. **Overall:** Overall risk of bias was judged as some concerns because at least one domain had some concerns and no domain was judged at high risk.								
**A056**	**Study/report:** de Souza et al., 2017 [9] **Comparison:** Superset vs traditional **Specific result:** Seated-row repetitions in sets 1–3 **Timepoint:** End of acute session **Reported result:** superset [8.0 ± 0.0; 6.2 ± 0.4; 5.3 ± 0.4]; traditional [8.0 ± 0.0; 6.8 ± 0.4; 5.9 ± 0.7] repetitions per set	**SC**	**SC**	**L**	**L**	**SC**	**SC**	**SC**
**Support for judgment. D1:** Randomization was reported, but sequence generation and/or allocation concealment were insufficiently described; baseline comparability by first-period sequence was not adequately reported. Evidence locator: PDF p.3, PDF p.4. **S:** The balance of intervention sequences and/or handling of period effects was insufficiently reported. The washout interval was probably adequate for an acute resistance-training response, but recovery before later periods was not directly verified. Evidence locator: PDF p.3, PDF p.4. **D2:** The intervention conditions were distinguishable to participants and personnel, but procedures were standardized and no trial-context deviations likely to affect the result were identified. The analysis retained the assigned condition or within-participant crossover comparison. Evidence locator: PDF pp.3-4, PDF p.4. **D3:** All or nearly all randomized participants appeared to contribute data for this specific result, with no meaningful missing outcome data identified. Evidence locator: PDF p.2, PDF p.4. **D4:** The result depended on counting technically valid repetitions and/or determining repetition failure by staff who could identify the intervention. Standardized technique and procedures reduced, but did not eliminate, potential measurement influence. Evidence locator: PDF pp.3-4, PDF p.4, PDF p.3. **D5:** No time-stamped trial registration, protocol, or statistical analysis plan was available to verify that the selected measure and analysis were prespecified before results were known. No direct selective reporting was identified, but selection among eligible measurements or analyses could not be excluded. Evidence locator: PDF pp.2-7, PDF p.4, PDF p.2. **Overall:** Overall risk of bias was judged as some concerns because at least one domain had some concerns and no domain was judged at high risk.								
**A057**	**Study/report:** de Souza et al., 2017 [9]	**SC**	**SC**	**L**	**L**	**SC**	**SC**	**SC**

ID	Result identification	D1	S	D2	D3	D4	D5	Overall
	**Comparison:** Superset vs traditional **Specific result:** Bench-press repetitions in sets 1–3 **Timepoint:** End of acute session **Reported result:** superset [7.9 ± 0.3; 6.5 ± 0.7; 5.2 ± 1.0]; traditional [8.0 ± 0.0; 6.8 ± 0.4; 5.9 ± 0.7] repetitions per set							
**Support for judgment. D1:** Randomization was reported, but sequence generation and/or allocation concealment were insufficiently described; baseline comparability by first-period sequence was not adequately reported. Evidence locator: PDF p.3, PDF p.4. **S:** The balance of intervention sequences and/or handling of period effects was insufficiently reported. The washout interval was probably adequate for an acute resistance-training response, but recovery before later periods was not directly verified. Evidence locator: PDF p.3, PDF p.4. **D2:** The intervention conditions were distinguishable to participants and personnel, but procedures were standardized and no trial-context deviations likely to affect the result were identified. The analysis retained the assigned condition or within-participant crossover comparison. Evidence locator: PDF pp.3-4, PDF p.4. **D3:** All or nearly all randomized participants appeared to contribute data for this specific result, with no meaningful missing outcome data identified. Evidence locator: PDF p.2, PDF p.4. **D4:** The result depended on counting technically valid repetitions and/or determining repetition failure by staff who could identify the intervention. Standardized technique and procedures reduced, but did not eliminate, potential measurement influence. Evidence locator: PDF pp.3-4, PDF p.4, PDF p.3. **D5:** No time-stamped trial registration, protocol, or statistical analysis plan was available to verify that the selected measure and analysis were prespecified before results were known. No direct selective reporting was identified, but selection among eligible measurements or analyses could not be excluded. Evidence locator: PDF pp.2-7, PDF p.4, PDF p.2. **Overall:** Overall risk of bias was judged as some concerns because at least one domain had some concerns and no domain was judged at high risk.								
**A059**	**Study/report:** Yamazaki et al., 2025 [44] **Comparison:** Alternating vs 4-min traditional **Specific result:** Bench-press total repetitions **Timepoint:** End of acute session **Reported result:** alternating 24.9 ± 5.2; 4-minute traditional 21.8 ± 4.2 repetitions	**SC**	**SC**	**L**	**L**	**SC**	**SC**	**SC**

<table>
<thead>
<tr><th>ID</th><th>Result identification</th><th>D1</th><th>S</th><th>D2</th><th>D3</th><th>D4</th><th>D5</th><th>Overall</th></tr>
</thead>
<tbody>
<tr><td colspan="9">Support for judgment. D1: Randomization was reported, but sequence generation and/or allocation concealment were insufficiently described; baseline comparability by first-period sequence was not adequately reported. Evidence locator: PDF p.2.
S: The balance of intervention sequences and/or handling of period effects was insufficiently reported. The washout interval was probably adequate for an acute resistance-training response, but recovery before later periods was not directly verified. Evidence locator: PDF p.2, PDF p.3.
D2: The intervention conditions were distinguishable to participants and personnel, but procedures were standardized and no trial-context deviations likely to affect the result were identified. The analysis retained the assigned condition or within-participant crossover comparison. Evidence locator: PDF pp.2-3, PDF pp.2-4.
D3: All or nearly all randomized participants appeared to contribute data for this specific result, with no meaningful missing outcome data identified. Evidence locator: PDF p.1, PDF p.3.
D4: The result depended on counting technically valid repetitions and/or determining repetition failure by staff who could identify the intervention. Standardized technique and procedures reduced, but did not eliminate, potential measurement influence. Evidence locator: PDF p.3, PDF pp.2-3.
D5: No time-stamped trial registration, protocol, or statistical analysis plan was available to verify that the selected measure and analysis were prespecified before results were known. No direct selective reporting was identified, but selection among eligible measurements or analyses could not be excluded. Evidence locator: PDF pp.1-6, PDF p.2, PDF p.3.
Overall: Overall risk of bias was judged as some concerns because at least one domain had some concerns and no domain was judged at high risk.</td></tr>
<tr><td>A060</td><td>Study/report: Yamazaki et al., 2025 [44]
Comparison: Alternating vs 4-min traditional
Specific result: Back-squat total repetitions
Timepoint: End of acute session
Reported result: alternating 27.2 ± 8.5; 4-minute traditional 22.6 ± 5.7 repetitions</td><td>SC</td><td>SC</td><td>L</td><td>L</td><td>SC</td><td>SC</td><td>SC</td></tr>
<tr><td colspan="9">Support for judgment. D1: Randomization was reported, but sequence generation and/or allocation concealment were insufficiently described; baseline comparability by first-period sequence was not adequately reported. Evidence locator: PDF p.2.
S: The balance of intervention sequences and/or handling of period effects was insufficiently reported. The washout interval was probably adequate for an acute resistance-training response, but recovery before later periods was not directly verified. Evidence locator: PDF p.2, PDF p.3.
D2: The intervention conditions were distinguishable to participants and personnel, but procedures were standardized and no trial-context deviations likely to affect the result were identified. The analysis retained the assigned condition or within-participant crossover comparison. Evidence locator: PDF pp.2-3, PDF pp.2-4.
D3: All or nearly all randomized participants appeared to contribute data for this specific result, with no meaningful missing outcome data identified. Evidence locator: PDF p.1, PDF p.3.
D4: The result depended on counting technically valid repetitions and/or determining repetition failure by staff who could identify the intervention. Standardized technique and procedures reduced, but did not eliminate, potential measurement influence. Evidence locator: PDF p.3, PDF pp.2-3.
D5: No time-stamped trial registration, protocol, or statistical analysis plan was available to verify that the selected measure and analysis were prespecified before results were known. No direct selective reporting was identified, but selection among eligible measurements or analyses could not be excluded. Evidence locator: PDF pp.1-6, PDF p.2, PDF p.3.
Overall: Overall risk of bias was judged as some concerns because at least one domain had some concerns and no domain was judged at high risk.</td></tr>
</tbody>
</table>

ID	Result identification	D1	S	D2	D3	D4	D5	Overall
A064	**Study/report:** Yamazaki et al., 2025 [44] **Comparison:** Alternating vs 8-min traditional **Specific result:** Bench-press total repetitions **Timepoint:** End of acute session **Reported result:** alternating 24.9 ± 5.2; 8-minute traditional 28.3 ± 6.3 repetitions	SC	SC	L	L	SC	SC	SC

Support for judgment. D1: Randomization was reported, but sequence generation and/or allocation concealment were insufficiently described; baseline comparability by first-period sequence was not adequately reported. Evidence locator: PDF p.2.
S: The balance of intervention sequences and/or handling of period effects was insufficiently reported. The washout interval was probably adequate for an acute resistance-training response, but recovery before later periods was not directly verified. Evidence locator: PDF p.2, PDF p.3.
D2: The intervention conditions were distinguishable to participants and personnel, but procedures were standardized and no trial-context deviations likely to affect the result were identified. The analysis retained the assigned condition or within-participant crossover comparison. Evidence locator: PDF pp.2-3, PDF pp.2-4.
D3: All or nearly all randomized participants appeared to contribute data for this specific result, with no meaningful missing outcome data identified. Evidence locator: PDF p.1, PDF p.3.
D4: The result depended on counting technically valid repetitions and/or determining repetition failure by staff who could identify the intervention. Standardized technique and procedures reduced, but did not eliminate, potential measurement influence. Evidence locator: PDF p.3, PDF pp.2-3.
D5: No time-stamped trial registration, protocol, or statistical analysis plan was available to verify that the selected measure and analysis were prespecified before results were known. No direct selective reporting was identified, but selection among eligible measurements or analyses could not be excluded. Evidence locator: PDF pp.1-6, PDF p.2, PDF p.3.
Overall: Overall risk of bias was judged as some concerns because at least one domain had some concerns and no domain was judged at high risk.

ID	Result identification	D1	S	D2	D3	D4	D5	Overall
A065	**Study/report:** Yamazaki et al., 2025 [44] **Comparison:** Alternating vs 8-min traditional **Specific result:** Back-squat total repetitions **Timepoint:** End of acute session **Reported result:** alternating 27.2 ± 8.5;	SC	SC	L	L	SC	SC	SC

ID	Result identification	D1	S	D2	D3	D4	D5	Overall
	8-minute traditional 28.0 ± 4.2 repetitions							

Support for judgment. D1: Randomization was reported, but sequence generation and/or allocation concealment were insufficiently described; baseline comparability by first-period sequence was not adequately reported. Evidence locator: PDF p.2.
S: The balance of intervention sequences and/or handling of period effects was insufficiently reported. The washout interval was probably adequate for an acute resistance-training response, but recovery before later periods was not directly verified. Evidence locator: PDF p.2, PDF p.3.
D2: The intervention conditions were distinguishable to participants and personnel, but procedures were standardized and no trial-context deviations likely to affect the result were identified. The analysis retained the assigned condition or within-participant crossover comparison. Evidence locator: PDF pp.2-3, PDF pp.2-4.
D3: All or nearly all randomized participants appeared to contribute data for this specific result, with no meaningful missing outcome data identified. Evidence locator: PDF p.1, PDF p.3.
D4: The result depended on counting technically valid repetitions and/or determining repetition failure by staff who could identify the intervention. Standardized technique and procedures reduced, but did not eliminate, potential measurement influence. Evidence locator: PDF p.3, PDF pp.2-3.
D5: No time-stamped trial registration, protocol, or statistical analysis plan was available to verify that the selected measure and analysis were prespecified before results were known. No direct selective reporting was identified, but selection among eligible measurements or analyses could not be excluded. Evidence locator: PDF pp.1-6, PDF p.2, PDF p.3.
Overall: Overall risk of bias was judged as some concerns because at least one domain had some concerns and no domain was judged at high risk.

ID	Result identification	D1	S	D2	D3	D4	D5	Overall
**A004**	**Study/report:** Peña García-Orea et al., 2022 [13] **Comparison:** Antagonist set vs traditional **Specific result:** Squat total repetitions across four load sessions **Timepoint:** End of acute session **Reported result:** Traditional 90.8 ± 27.0; antagonist 73.1 ± 18.1	**SC**	**N/A**	**L**	**L**	**L**	**SC**	**SC**

Support for judgment. D1: Randomization was reported, but sequence generation and/or allocation concealment were insufficiently described; baseline comparability by randomized group was not adequately reported.
S: Not applicable to this parallel-group trial.
D2: The intervention conditions were distinguishable to participants and personnel, but procedures were standardized and no trial-context deviations likely to affect the result were identified. The analysis retained the assigned condition or within-participant crossover comparison. Evidence locator: PDF pp.2-3, PDF p.2.
D3: All or nearly all randomized participants appeared to contribute data for this specific result, with no meaningful missing outcome data identified.
D4: Repetitions were determined using objective velocity-transducer data and a prespecified velocity-loss criterion applied similarly across groups; unblinded supervision was unlikely to alter the recorded count.
D5: No time-stamped trial registration, protocol, or statistical analysis plan was available to verify that the selected measure and analysis were prespecified before results were known. No direct selective reporting was identified, but selection among eligible measurements or analyses could not be excluded. Evidence locator: PDF p.11.
Overall: Overall risk of bias was judged as some concerns because at least one domain had some concerns and no domain was judged at high risk.

ID	Result identification	D1	S	D2	D3	D4	D5	Overall
A005	**Study/report:** Peña García-Orea et al., 2022 [13] **Comparison:** Antagonist set vs traditional **Specific result:** Bench-press total repetitions across four load sessions **Timepoint:** End of acute session **Reported result:** Traditional 75.1 ± 23.9; antagonist 73.5 ± 12.9	SC	N/A	L	L	L	SC	SC

Support for judgment. D1: Randomization was reported, but sequence generation and/or allocation concealment were insufficiently described; baseline comparability by randomized group was not adequately reported.
S: Not applicable to this parallel-group trial.
D2: The intervention conditions were distinguishable to participants and personnel, but procedures were standardized and no trial-context deviations likely to affect the result were identified. The analysis retained the assigned condition or within-participant crossover comparison. Evidence locator: PDF pp.2-3, PDF p.2.
D3: All or nearly all randomized participants appeared to contribute data for this specific result, with no meaningful missing outcome data identified.
D4: Repetitions were determined using objective velocity-transducer data and a prespecified velocity-loss criterion applied similarly across groups; unblinded supervision was unlikely to alter the recorded count.
D5: No time-stamped trial registration, protocol, or statistical analysis plan was available to verify that the selected measure and analysis were prespecified before results were known. No direct selective reporting was identified, but selection among eligible measurements or analyses could not be excluded. Evidence locator: PDF p.11.
Overall: Overall risk of bias was judged as some concerns because at least one domain had some concerns and no domain was judged at high risk.

Note. D1 = bias arising from the randomization process; S = bias arising from period and carryover effects; D2 = bias due to deviations from intended interventions; D3 = bias due to missing outcome data; D4 = bias in measurement of the outcome; D5 = bias in selection of the reported result. L = low risk of bias; SC = some concerns; H = high risk of bias; N/A = not applicable. Support for judgment is a concise English synthesis of the final consensus rationale. Evidence locators are retained where available. Complete consensus responses to the RoB 2 signalling questions and the full source-located rationale should be supplied with the final consensus workbook as supplementary data.

Table S3. (C) Detailed Result-Level RoB 2 Assessments for Training Efficiency Outcomes.

ID	Result identification	D1	S	D2	D3	D4	D5	Overall
**A020**	**Study/report:** Paz et al., 2019b [17] **Comparison:** Paired set vs traditional **Specific result:** Volume load per minute **Timepoint:** End of acute session **Reported result:** Traditional 191.7 ± 79.1; paired set 199.0 ± 85.0	**SC**	**SC**	**L**	**L**	**SC**	**SC**	**SC**
**Support for judgment. D1:** Randomization was reported, but sequence generation and/or allocation concealment were insufficiently described; baseline comparability by first-period sequence was not adequately reported. Evidence locator: PDF p.1, PDF p.2, PDF p.3. **S:** The balance of intervention sequences and/or handling of period effects was insufficiently reported. The washout interval was probably adequate for an acute resistance-training response, but recovery before later periods was not directly verified. Evidence locator: PDF p.3, PDF p.5, PDF p.4. **D2:** The intervention conditions were distinguishable to participants and personnel, but procedures were standardized and no trial-context deviations likely to affect the result were identified. The analysis retained the assigned condition or within-participant crossover comparison. Evidence locator: PDF p.3, PDF p.4, PDF p.6. **D3:** All or nearly all randomized participants appeared to contribute data for this specific result, with no meaningful missing outcome data identified. Evidence locator: PDF p.1, PDF p.5, PDF p.6. **D4:** Efficiency incorporated volume load derived from valid-repetition counts. Because staff could identify the intervention, minor influence on repetition/failure judgments could affect the derived metric despite standardized procedures. Evidence locator: PDF p.6, PDF p.4, PDF p.9. **D5:** No time-stamped trial registration, protocol, or statistical analysis plan was available to verify that the selected measure and analysis were prespecified before results were known. No direct selective reporting was identified, but selection among eligible measurements or analyses could not be excluded. Evidence locator: PDF p.6, PDF p.5. **Overall:** Overall risk of bias was judged as some concerns because at least one domain had some concerns and no domain was judged at high risk.								
**A023**	**Study/report:** Paz et al., 2019b [17] **Comparison:** Superset vs traditional **Specific result:** Volume load per minute **Timepoint:** End of acute session **Reported result:** Traditional 191.7 ± 79.1; superset 193.3 ± 70.2	**SC**	**SC**	**L**	**L**	**SC**	**SC**	**SC**

ID	Result identification	D1	S	D2	D3	D4	D5	Overall
**Support for judgment. D1:** Randomization was reported, but sequence generation and/or allocation concealment were insufficiently described; baseline comparability by first-period sequence was not adequately reported. Evidence locator: PDF p.1, PDF p.2, PDF p.3.<br>**S:** The balance of intervention sequences and/or handling of period effects was insufficiently reported. The washout interval was probably adequate for an acute resistance-training response, but recovery before later periods was not directly verified. Evidence locator: PDF p.3, PDF p.5, PDF p.4.<br>**D2:** The intervention conditions were distinguishable to participants and personnel, but procedures were standardized and no trial-context deviations likely to affect the result were identified. The analysis retained the assigned condition or within-participant crossover comparison. Evidence locator: PDF p.3, PDF p.4, PDF p.6.<br>**D3:** All or nearly all randomized participants appeared to contribute data for this specific result, with no meaningful missing outcome data identified. Evidence locator: PDF p.1, PDF p.5, PDF p.6.<br>**D4:** Efficiency incorporated volume load derived from valid-repetition counts. Because staff could identify the intervention, minor influence on repetition/failure judgments could affect the derived metric despite standardized procedures. Evidence locator: PDF p.6, PDF p.4, PDF p.9.<br>**D5:** No time-stamped trial registration, protocol, or statistical analysis plan was available to verify that the selected measure and analysis were prespecified before results were known. No direct selective reporting was identified, but selection among eligible measurements or analyses could not be excluded. Evidence locator: PDF p.6, PDF p.5.<br>**Overall:** Overall risk of bias was judged as some concerns because at least one domain had some concerns and no domain was judged at high risk.								
A026	**Study/report:** Robbins et al., 2010a [18]<br>**Comparison:** Complex paired set vs traditional<br>**Specific result:** Bench-pull volume load per minute<br>**Timepoint:** End of acute session<br>**Reported result:** Complex 103.47 kg/min; traditional 54.71 kg/min	SC	L	L	L	SC	SC	SC
**Support for judgment. D1:** Randomization was reported, but sequence generation and/or allocation concealment were insufficiently described; baseline comparability by first-period sequence was not adequately reported. Evidence locator: PDF p.2, PDF p.3.<br>**S:** Counterbalancing/randomized order and the washout interval supported a low risk of period or carryover bias; no material carryover problem was identified, although sequence counts or pre-period recovery checks were not always fully reported. Evidence locator: PDF p.2, PDF p.3.<br>**D2:** The intervention conditions were distinguishable to participants and personnel, but procedures were standardized and no trial-context deviations likely to affect the result were identified. The analysis retained the assigned condition or within-participant crossover comparison. Evidence locator: PDF p.5-6, PDF p.3, PDF p.6.<br>**D3:** All or nearly all randomized participants appeared to contribute data for this specific result, with no meaningful missing outcome data identified. Evidence locator: PDF p.2, PDF p.5.<br>**D4:** Efficiency incorporated volume load derived from valid-repetition counts. Because staff could identify the intervention, minor influence on repetition/failure judgments could affect the derived metric despite standardized procedures. Evidence locator: PDF p.5.<br>**D5:** No time-stamped trial registration, protocol, or statistical analysis plan was available to verify that the selected measure and analysis were prespecified before results were known. No direct selective reporting was identified, but selection among eligible measurements or analyses could not be excluded. Evidence locator: PDF p.5, PDF p.6, PDF p.2.<br>**Overall:** Overall risk of bias was judged as some concerns because at least one domain had some concerns and no domain was judged at high risk.								

ID	Result identification	D1	S	D2	D3	D4	D5	Overall
A030	**Study/report:** Robbins et al., 2010b [19] **Comparison:** Agonist–antagonist paired set vs traditional **Specific result:** Bench-pull volume load per minute **Timepoint:** End of acute session **Reported result:** Paired set 89.5 kg/min; traditional 73.8 kg/min	SC	L	L	L	SC	SC	SC

Support for judgment. D1: Randomization was reported, but sequence generation and/or allocation concealment were insufficiently described; baseline comparability by first-period sequence was not adequately reported. Evidence locator: PDF p.3.
S: Counterbalancing/randomized order and the washout interval supported a low risk of period or carryover bias; no material carryover problem was identified, although sequence counts or pre-period recovery checks were not always fully reported. Evidence locator: PDF p.3, PDF p.4.
D2: The intervention conditions were distinguishable to participants and personnel, but procedures were standardized and no trial-context deviations likely to affect the result were identified. The analysis retained the assigned condition or within-participant crossover comparison. Evidence locator: PDF p.3, PDF pp.3-5, PDF p.5.
D3: All or nearly all randomized participants appeared to contribute data for this specific result, with no meaningful missing outcome data identified. Evidence locator: PDF p.4, PDF p.5.
D4: Efficiency incorporated volume load derived from valid-repetition counts. Because staff could identify the intervention, minor influence on repetition/failure judgments could affect the derived metric despite standardized procedures. Evidence locator: PDF p.5, PDF p.6.
D5: No time-stamped trial registration, protocol, or statistical analysis plan was available to verify that the selected measure and analysis were prespecified before results were known. No direct selective reporting was identified, but selection among eligible measurements or analyses could not be excluded. Evidence locator: PDF pp.1-9, PDF p.5, PDF p.6.
Overall: Overall risk of bias was judged as some concerns because at least one domain had some concerns and no domain was judged at high risk.

ID	Result identification	D1	S	D2	D3	D4	D5	Overall
**A031**	**Study/report:** Robbins et al., 2010b [19] **Comparison:** Agonist–antagonist paired set vs traditional **Specific result:** Bench-press volume load per minute **Timepoint:** End of acute session **Reported result:** Paired set 93.0 kg/min; traditional 73.1 kg/min	**SC**	**L**	**L**	**L**	**SC**	**SC**	**SC**

Support for judgment. D1: Randomization was reported, but sequence generation and/or allocation concealment were insufficiently described; baseline comparability by first-period sequence was not adequately reported. Evidence locator: PDF p.3.

S: Counterbalancing/randomized order and the washout interval supported a low risk of period or carryover bias; no material carryover problem was identified, although sequence counts or pre-period recovery checks were not always fully reported. Evidence locator: PDF p.3, PDF p.4.

D2: The intervention conditions were distinguishable to participants and personnel, but procedures were standardized and no trial-context deviations likely to affect the result were identified. The analysis retained the assigned condition or within-participant crossover comparison. Evidence locator: PDF p.3, PDF pp.3-5, PDF p.5.

D3: All or nearly all randomized participants appeared to contribute data for this specific result, with no meaningful missing outcome data identified. Evidence locator: PDF p.4, PDF p.5.

D4: Efficiency incorporated volume load derived from valid-repetition counts. Because staff could identify the intervention, minor influence on repetition/failure judgments could affect the derived metric despite standardized procedures. Evidence locator: PDF p.5, PDF p.6.

D5: No time-stamped trial registration, protocol, or statistical analysis plan was available to verify that the selected measure and analysis were prespecified before results were known. No direct selective reporting was identified, but selection among eligible measurements or analyses could not be excluded. Evidence locator: PDF pp.1-9, PDF p.5, PDF p.6.

Overall: Overall risk of bias was judged as some concerns because at least one domain had some concerns and no domain was judged at high risk.

ID	Result identification	D1	S	D2	D3	D4	D5	Overall
**A035**	**Study/report:** Robbins et al., 2010c [20] **Comparison:** Paired set vs traditional **Specific result:** Bench-pull volume load per minute **Timepoint:** End of acute session **Reported result:** Paired set 106.06 kg/min; traditional 55.27 kg/min	**SC**	**L**	**L**	**L**	**SC**	**SC**	**SC**

Support for judgment. D1: Randomization was reported, but sequence generation and/or allocation concealment were insufficiently described; baseline comparability by first-period sequence was not adequately reported. Evidence locator: PDF p.2, PDF p.3.
S: Counterbalancing/randomized order and the washout interval supported a low risk of period or carryover bias; no material carryover problem was identified, although sequence counts or pre-period recovery checks were not always fully reported. Evidence locator: PDF p.2, PDF p.4, PDF p.3.
D2: The intervention conditions were distinguishable to participants and personnel, but procedures were standardized and no trial-context deviations likely to affect the result were identified. The analysis retained the assigned condition or within-participant crossover comparison. Evidence locator: PDF pp.2-3, PDF pp.2-5, PDF p.6.
D3: All or nearly all randomized participants appeared to contribute data for this specific result, with no meaningful missing outcome data identified. Evidence locator: PDF p.3, PDF p.6.
D4: Efficiency incorporated volume load derived from valid-repetition counts. Because staff could identify the intervention, minor influence on repetition/failure judgments could affect the derived metric despite standardized procedures. Evidence locator: PDF p.6.
D5: No time-stamped trial registration, protocol, or statistical analysis plan was available to verify that the selected measure and analysis were prespecified before results were known. No direct selective reporting was identified, but selection among eligible measurements or analyses could not be excluded. Evidence locator: PDF pp.1-9, PDF p.6, PDF p.1.
Overall: Overall risk of bias was judged as some concerns because at least one domain had some concerns and no domain was judged at high risk.

ID	Result identification	D1	S	D2	D3	D4	D5	Overall
**A036**	**Study/report:** Robbins et al., 2010c [20] **Comparison:** Paired set vs traditional **Specific result:** Bench-press volume load per minute **Timepoint:** End of acute session **Reported result:** Paired set 103.96 kg/min; traditional 55.37 kg/min	**SC**	**L**	**L**	**L**	**SC**	**SC**	**SC**

ID	Result identification	D1	S	D2	D3	D4	D5	Overall
**Support for judgment. D1:** Randomization was reported, but sequence generation and/or allocation concealment were insufficiently described; baseline comparability by first-period sequence was not adequately reported. Evidence locator: PDF p.2, PDF p.3.<br>**S:** Counterbalancing/randomized order and the washout interval supported a low risk of period or carryover bias; no material carryover problem was identified, although sequence counts or pre-period recovery checks were not always fully reported. Evidence locator: PDF p.2, PDF p.4, PDF p.3.<br>**D2:** The intervention conditions were distinguishable to participants and personnel, but procedures were standardized and no trial-context deviations likely to affect the result were identified. The analysis retained the assigned condition or within-participant crossover comparison. Evidence locator: PDF pp.2-3, PDF pp.2-5, PDF p.6.<br>**D3:** All or nearly all randomized participants appeared to contribute data for this specific result, with no meaningful missing outcome data identified. Evidence locator: PDF p.3, PDF p.6.<br>**D4:** Efficiency incorporated volume load derived from valid-repetition counts. Because staff could identify the intervention, minor influence on repetition/failure judgments could affect the derived metric despite standardized procedures. Evidence locator: PDF p.6.<br>**D5:** No time-stamped trial registration, protocol, or statistical analysis plan was available to verify that the selected measure and analysis were prespecified before results were known. No direct selective reporting was identified, but selection among eligible measurements or analyses could not be excluded. Evidence locator: PDF pp.1-9, PDF p.6, PDF p.1.<br>**Overall:** Overall risk of bias was judged as some concerns because at least one domain had some concerns and no domain was judged at high risk.								
**A038**	**Study/report:** Weakley et al., 2017 [21]<br>**Comparison:** Superset vs traditional<br>**Specific result:** Volume load per minute<br>**Timepoint:** End of acute session<br>**Reported result:** Superset 483.3 ± 106.3 kg/min; traditional 275.0 ± 52.3 kg/min	**SC**	**SC**	**L**	**L**	**L**	**SC**	**SC**
**Support for judgment. D1:** Randomization was reported, but sequence generation and/or allocation concealment were insufficiently described; baseline comparability by first-period sequence was not adequately reported. Evidence locator: PDF pp.2-3.<br>**S:** The balance of intervention sequences and/or handling of period effects was insufficiently reported. The washout interval was probably adequate for an acute resistance-training response, but recovery before later periods was not directly verified. Evidence locator: PDF pp.2-3, PDF p.5, PDF p.3.<br>**D2:** The intervention conditions were distinguishable to participants and personnel, but procedures were standardized and no trial-context deviations likely to affect the result were identified. The analysis retained the assigned condition or within-participant crossover comparison. Evidence locator: PDF pp.3-5, PDF pp.3-4, PDF p.1.<br>**D3:** All or nearly all randomized participants appeared to contribute data for this specific result, with no meaningful missing outcome data identified. Evidence locator: PDF p.1, PDF p.6.<br>**D4:** Efficiency was calculated from standardized volume-load and objectively recorded training-duration components using the same formula across conditions; condition awareness was unlikely to alter the derived value. Evidence locator: PDF p.5.<br>**D5:** No time-stamped trial registration, protocol, or statistical analysis plan was available to verify that the selected measure and analysis were prespecified before results were known. No direct selective reporting was identified, but selection among eligible measurements or analyses could not be excluded. Evidence locator: PDF pp.1-13, PDF p.5, PDF p.6.<br>**Overall:** Overall risk of bias was judged as some concerns because at least one domain had some concerns and no domain was judged at high risk.								

ID	Result identification	D1	S	D2	D3	D4	D5	Overall
**A040**	**Study/report:** Nasser et al., 2019 [43] **Comparison:** APS vs traditional **Specific result:** Author-reported training-efficiency analysis **Timepoint:** End of acute session **Reported result:**APS efficiency was significantly higher than TM (*p* = .005); exact condition means and SDs were not reported numerically	**SC**	**L**	**L**	**L**	**SC**	**SC**	**SC**

Support for judgment. D1: Randomization was reported, but sequence generation and/or allocation concealment were insufficiently described; baseline comparability by first-period sequence was not adequately reported. Evidence locator: PDF pp.18-19, PDF p.18.
S: Counterbalancing/randomized order and the washout interval supported a low risk of period or carryover bias; no material carryover problem was identified, although sequence counts or pre-period recovery checks were not always fully reported. Evidence locator: PDF pp.17-19, PDF pp.18-19.
D2: The intervention conditions were distinguishable to participants and personnel, but procedures were standardized and no trial-context deviations likely to affect the result were identified. The analysis retained the assigned condition or within-participant crossover comparison. Evidence locator: PDF pp.17-19, PDF pp.18-19, PDF p.21.
D3: All or nearly all randomized participants appeared to contribute data for this specific result, with no meaningful missing outcome data identified. Evidence locator: PDF p.17, PDF p.21.
D4: Efficiency incorporated volume load derived from valid-repetition counts. Because staff could identify the intervention, minor influence on repetition/failure judgments could affect the derived metric despite standardized procedures. Evidence locator: PDF pp.19.
D5: No time-stamped trial registration, protocol, or statistical analysis plan was available to verify that the selected measure and analysis were prespecified before results were known. No direct selective reporting was identified, but selection among eligible measurements or analyses could not be excluded. Evidence locator: PDF pp.17-22, PDF pp.19, PDF p.19.
Overall: Overall risk of bias was judged as some concerns because at least one domain had some concerns and no domain was judged at high risk.

ID	Result identification	D1	S	D2	D3	D4	D5	Overall
**A046**	**Study/report:** Nasser et al., 2019 [43] **Comparison:** PAL vs traditional **Specific result:** Author-reported training-efficiency analysis **Timepoint:** End of acute session **Reported result:** PAL efficiency was significantly higher than TM (p < .001); exact condition means and SDs were not reported numerically.	**SC**	**L**	**L**	**L**	**SC**	**SC**	**SC**

Support for judgment. D1: Randomization was reported, but sequence generation and/or allocation concealment were insufficiently described; baseline comparability by first-period sequence was not adequately reported. Evidence locator: PDF pp.18-19, PDF p.18.

S: Counterbalancing/randomized order and the washout interval supported a low risk of period or carryover bias; no material carryover problem was identified, although sequence counts or pre-period recovery checks were not always fully reported. Evidence locator: PDF pp.17-19, PDF pp.18-19.

D2: The intervention conditions were distinguishable to participants and personnel, but procedures were standardized and no trial-context deviations likely to affect the result were identified. The analysis retained the assigned condition or within-participant crossover comparison. Evidence locator: PDF pp.17-19, PDF pp.18-19, PDF p.21.

D3: All or nearly all randomized participants appeared to contribute data for this specific result, with no meaningful missing outcome data identified. Evidence locator: PDF p.17, PDF p.21.

D4: Efficiency incorporated volume load derived from valid-repetition counts. Because staff could identify the intervention, minor influence on repetition/failure judgments could affect the derived metric despite standardized procedures. Evidence locator: PDF pp.19.

D5: No time-stamped trial registration, protocol, or statistical analysis plan was available to verify that the selected measure and analysis were prespecified before results were known. No direct selective reporting was identified, but selection among eligible measurements or analyses could not be excluded. Evidence locator: PDF pp.17-22, PDF pp.19, PDF p.19.

Overall: Overall risk of bias was judged as some concerns because at least one domain had some concerns and no domain was judged at high risk.

Note. D1 = bias arising from the randomization process; S = bias arising from period and carryover effects; D2 = bias due to deviations from intended interventions; D3 = bias due to missing outcome data; D4 = bias in measurement of the outcome; D5 = bias in selection of the reported result. L = low risk of bias; SC = some concerns; H = high risk of bias; N/A = not applicable; APS = agonist–antagonist paired-set method; PAL = paired alternating upper–lower-limb method; TM = traditional method. Support for judgment is a concise English synthesis of the final consensus rationale. Evidence locators are retained where available.
